# Inhibition of the Hexosamine Biosynthetic Pathway by targeting PGM3 causes breast cancer growth arrest and apoptosis

**DOI:** 10.1038/s41419-018-0405-4

**Published:** 2018-03-07

**Authors:** Francesca Ricciardiello, Giuseppina Votta, Roberta Palorini, Isabella Raccagni, Laura Brunelli, Alice Paiotta, Francesca Tinelli, Giuseppe D’Orazio, Silvia Valtorta, Luca De Gioia, Roberta Pastorelli, Rosa Maria Moresco, Barbara La Ferla, Ferdinando Chiaradonna

**Affiliations:** 1 Department of Biotechnology and Biosciences, University of Milano-Bicocca, Milan, 20126 Italy; 2 Institute of Molecular Bioimaging and Physiology (IBFM), CNR, Segrate, 20090 Italy; 3 Environmental Health Sciences Department, Istituto di Ricerche Farmacologiche Mario Negri, Milan, 20156 Italy; 4 School of Medicine and Surgery, University of Milan-Bicocca, Monza, 20900 Italy

## Abstract

Cancer aberrant *N*- and *O*-linked protein glycosylation, frequently resulting from an augmented flux through the Hexosamine Biosynthetic Pathway (HBP), play different roles in tumor progression. However, the low specificity and toxicity of the existing HBP inhibitors prevented their use for cancer treatment. Here we report the preclinical evaluation of FR054, a novel inhibitor of the HBP enzyme PGM3, with a remarkable anti-breast cancer effect. In fact, FR054 induces in different breast cancer cells a dramatic decrease in cell proliferation and survival. In particular, in a model of Triple Negative Breast Cancer (TNBC) cells, MDA-MB-231, we show that these effects are correlated to FR054-dependent reduction of both *N*- and *O*-glycosylation level that cause also a strong reduction of cancer cell adhesion and migration. Moreover we show that impaired survival of cancer cells upon FR054 treatment is associated with the activation of the Unfolded Protein Response (UPR) and accumulation of intracellular ROS. Finally, we show that FR054 suppresses cancer growth in MDA-MB-231 xenograft mice, supporting the advantage of targeting HBP for therapeutic purpose and encouraging further investigation about the use of this small molecule as a promising compound for breast cancer therapy.

## Introduction

Glycans are the most abundant and complex group of molecules in living organisms. Frequently attached to proteins to form simple and complex glycoconjugates, in a process defined *N*-glycosylation (*N*-Glc*N*Ac) and *O*-Glc*N*Acylation (*O*-Glc*N*Ac), they regulate several aspects of protein function and participate in many key physiological processes including cellular adhesion, migration, growth, differentiation, signal transduction, receptor activation, and quality control of protein folding^1^. Glycoconjugates play also different roles in several steps of tumor progression regulating tumor proliferation, invasion, metastasis, and angiogenesis^2,3^. Recent findings suggest that aberrant glycosylation and its biosynthetic machinery is a promising target for antitumor drugs^4^.

**Fig. 1:**
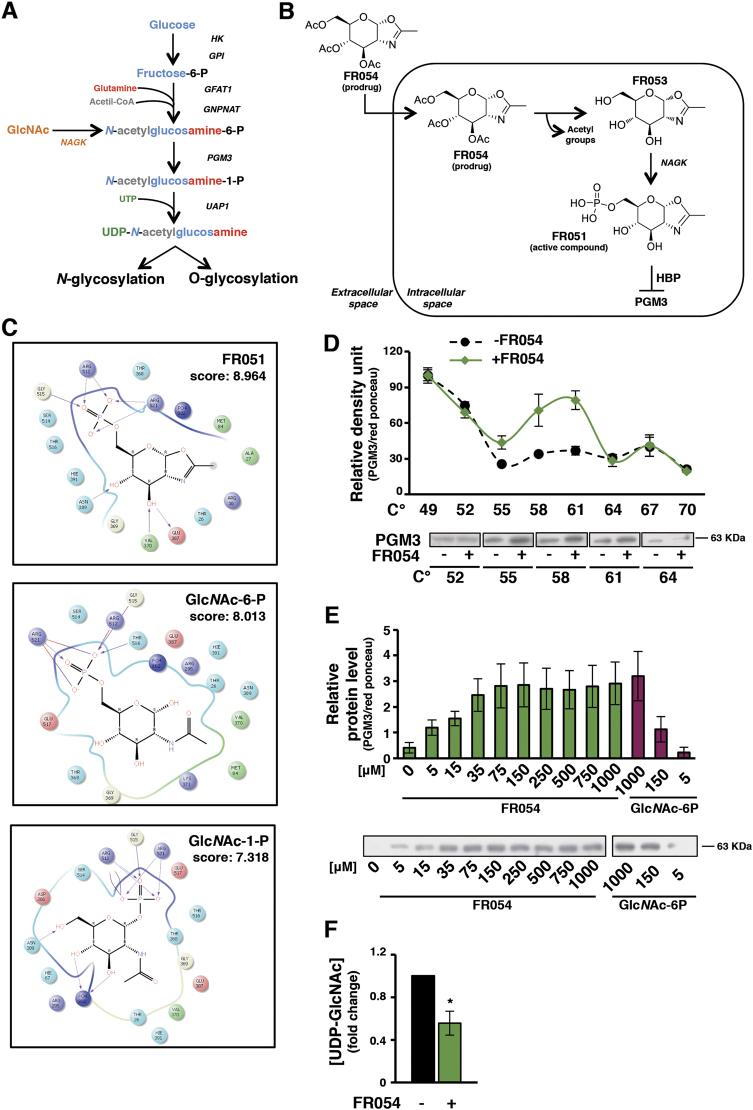
In silico and in vivo analysis reveal FR054 specificity for PGM3 enzyme. **a** Schematic representation of HBP. **b** Schematic drawing of the conversion of FR054 (prodrug) into FR051 (active compound). **c** Poses of the FR051 (upper), Glc*N*Ac-6-P (middle), and Glc*N*Ac-1-P (bottom) molecules in the catalytic cleft of PGM3 and their docking scores (kj/mol). **d** CETSA curves for PGM3 with FR054 measured in MDA-MB-231 cell extracts from 49 to 70 °C. **e** ITDRF_CETSA_ curves for FR054 and Glc*N*Ac-6-P in cell extracts at 58 °C. **f** HPLC quantification of UDP-Glc*N*Ac in cell extracts of MDA-MB-231 upon FR054 treatment. All data represent the average ± s.d.; *p < 0.05 (Student’s *t*-test; comparison with FR054-not-treated sample); *N* = 3

Glycan structures and types depend on gene expression, the activities of several enzymes, and the availability of the main donor substrate, uridine diphosphate *N*-Acetylglucosamine (UDP-Glc*N*Ac). The donor substrate is derived from glucose (Glc), glutamine, AcetylCoA, and uridine-5′-triphosphate as well as from intracellular degradation of glycoconjugates in lysosomes (salvage pathway) through the metabolic route termed Hexosamine Biosynthetic Pathway (HBP) (Fig. 1a).

Remarkably, previous work linked tumor development with an enhanced flux through the HBP^5,6^. In this regard, complex glycans synthesis, present in integrins and growth factor receptors, and protein *O*-Glc*N*Ac are limited by the intracellular amount of UDP-Glc*N*Ac^7,8^, suggesting that it may be possible to target cancer surface distribution of growth factor receptors and/or aberrant protein *O*-Glc*N*Ac by modulating HBP flux.

Despite the advances in structural and mechanistic features of enzymes involved in glycosylation, the existing inhibitors are not always specific and efficient, basically target only one of the two different HBP’s branches, and are too toxic to be developed for clinical use^9,10^. In this scenario, the identification of novel HBP inhibitors, capable of targeting both branches more effectively and in a selective manner, is an important challenge in cancer therapy.

Among the different enzymes involved in this pathway, we focused on the *N*-Acetylglucosamine-phosphate Mutase (PGM3) that catalyzes the conversion of *N*-Acetylglucosamine-6-phosphate (Glc*N*Ac-6-P) into *N*-Acetylglucosamine-1-phosphate (Glc*N*Ac-1-P). This enzyme has been chosen for different reasons: its modulation permits to control both *N*- and *O*-Glc*N*Ac; it is downstream to the salvage pathway; it is an unfavorable marker for breast cancer;^11^ mouse and human cells, in which PGM3 activity is reduced, show a decrease in HBP flux associated with the reduction of complex *N*-glycans and of *O*-Glc*N*Ac level^12–14^. We aimed to develop a PGM3 competitive inhibitor by modifying the chemical structure of Glc*N*Ac and generating substrate analogs. We hypothesized that treating cells with a cell-permeable glycomimetic biosynthetic precursor (prodrug), the HBP could generate in cells a PGM3 inhibitor (active compound) upon phosphorylation by *N*-Acetylglucosamine kinase (NAGK), the kinase assigned to Glc*N*Ac entry into HBP pathway through the salvage pathway (Fig. 1b).

Recent studies reported an association between the tumorigenic potential, metastasis, and chemoresistance of several types of breast cancer cells and tumors, among which the Triple Negative Breast Cancer (TNBC), and the alteration of their membrane glycans composition and ramification as well as of their level of protein *O*-Glc*N*Ac^4,15,16^. Since an HBP inhibitor could represent a novel treatment option, especially for TNBC, here we demonstrated the antitumor effect and the action mechanism of a novel HBP inhibitor in both in vitro and in vivo breast cancer models.

## Results

### Design, computational analysis, and synthesis of molecules able to attenuate HBP flux by targeting PGM3 enzyme

Different studies have demonstrated that the available HBP inhibitors, Azaserine (Aza) and 6-diazo-5-oxo-l-norleucine (DON), being glutamine analogs, are not specific^17^. However, clinical studies with both compounds showed certain antitumor activity, highlighting the advantage of the approach^18^.

To identify a potential selective inhibitor of HBP’s enzyme PGM3, we designed a series of compounds resembling the structure of the substrate and presenting a modification in one of the key positions involved in the catalysis^19^.

Among them we identified as good candidate the glycomimetic compound named FR051 (active compound) characterized by a phosphorylated OH group at position C-6 and by a fused oxazoline ring between position C-1 and C-2 (Fig. 1b). Docking calculations of its interaction with the catalytic cleft of PGM3 indicated that FR051 had a higher score as compared to the natural substrate (Glc*N*Ac-6-P) and product (Glc*N*Ac-1-P) supporting the potential interaction of FR051 with PGM3 (Fig. 1c). Then we designed and synthesized, according to a procedure previously described^20^, two prodrugs, named FR053 and FR054, whose main difference was the presence of three acetyl groups on C-3,4,6 OH in FR054 as compared to FR053, that improved FR054 cell permeability (structure, ^1^H Nuclear Magnetic Resonance (NMR) analysis, and cLogP are shown in Supplementary Figure 1). To prove the interaction between FR054 and PGM3 in cells, a Cellular Thermal Shift Assay (CETSA) was employed. Compared with DMSO, FR054 markedly increased the accumulation of PGM3 in the soluble fraction at the temperatures examined (Fig. 1d). Importantly, FR054 did not change the thermal stability of another HBP-specific protein, UDP-*N*-Acetylglucosamine Pyrophosphorylase-1 (UAP1), as well as of Vinculin (Supplementary Figure 2) confirming the specific effect of FR054 on PGM3. Then, we tested the dose-response of FR054 on the stability of PGM3 to heating. As shown in Fig. 1e, FR054 enhanced the stability of PGM3 protein in a dose-dependent manner (Fig. 1e) and with a more potent binding than the natural substrate Glc*N*Ac-6-P, because at the lowest doses used, 5 and 150 μM, PGM3 protein was more stable. Thus, PGM3 is confirmed as the direct binding target of FR054 in cell lysates. The inhibitory activity of FR054 on PGM3 was indirectly evaluated by HPLC measurement of UDP-Glc*N*Ac concentration in cell extracts. FR054 led to an UDP-Glc*N*Ac reduction of almost 50% in 30 min (Fig. 1f). Altogether these data suggest that FR054 directly interacts with intracellular PGM3 and that such interaction leads to PGM3 activity inhibition.

### FR054 induces an early proliferation arrest followed by a marked cell death increase in breast cancer cells

**Fig. 2:**
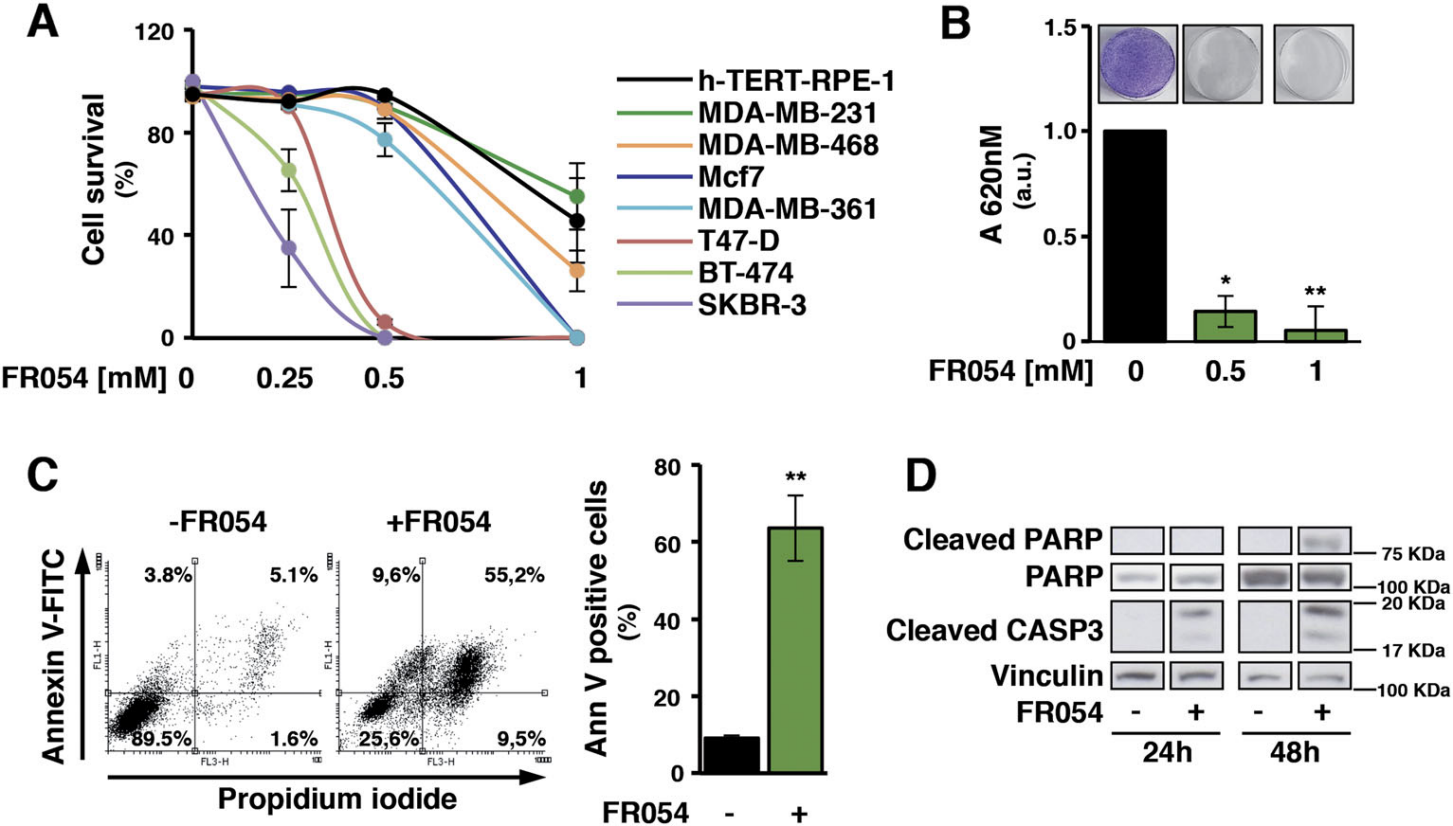
FR054 treatment leads to cell growth arrest and apoptosis of several breast cancer cells characterized by a diverse genetic background. **a** Viability of breast cancer cells and hTERT-RPE-1 upon 48 h treatment with different doses of FR054. **b** Colony formation of MDA-MB-231 cell treated with vehicle (medium) and two different doses of FR054 (0.5 and 1 mM) for 48 h. Representative images of the plates are shown (upper part of the panel). **c** Cell death evaluation by using FACS analysis of Annexin V-FITC (Ann V) and propidium iodide staining in MDA-MB-231 cells treated with 1 mM FR054 for 48 h. Quantification of Ann V positive cells is reported in the histogram. **d** Western blot analysis of PARP cleavage and Caspase3 activation in MDA-MB-231 cells treated for 48 h with 1 mM FR054. All data represent the average ± s.d.; *p < 0.05, **p < 0.01 (Student’s *t*-test; comparison with FR054-not-treated sample); *N* = 3

To examine the ability of FR054 to interfere with cancer cell proliferation and survival, as previously shown with other HBP and *N*-Glc*N*Ac inhibitors^21^, at first we examined the effect of 48 h FR054 treatment (from 250 μM to 1 mM) on seven different breast cancer cells. FR054 affected dose dependently their survival (Fig. 2a) and proliferation (Supplementary Figure 3A). Importantly six on seven cancer cells resulted more sensitive than non-transformed retinal pigmented epithelial cells (hTERT-RPE-1)^22,23^. TNBC cells, MDA-MB-231 and MDA-MB-468, had a lower sensitivity as compared to the other cancer cells. Nevertheless, at 72 h FR054 treatment, nearly 100% of TNBC cells appeared dead (Supplementary Figure 3B). Importantly, at 72 h the highest dose of low permeable compound, FR053, had no effect on MDA-MB-231 cell proliferation and viability, indicating that acetylation of FR054 is necessary for its cell penetration and effect (Supplementary Figure 3C). Since TNBCs are less sensible and difficult-to-treat, MDA-MB-231 cells were selected for further studies. Analysis of clonogenic activity in MDA-MB-231 cells treated 24 h with 0.5 and 1 mM FR054 revealed almost complete inhibition of colony formation (Fig. 2b). To correlate the described cell effects with the intracellular concentrations of the prodrug and the active compound, we evaluated the concentration by Liquid Chromatography/Mass Spectrometry (LC/MS) of the acetylated (FR054), the de-acetylated (FR053), and de-acetylated and phosphorylated (FR051) forms, indicative of the prodrug penetration and the subsequent transformation into the active compound, in MDA-MB-231 cells upon 24 h treatment with 1 mM FR054. Semiquantitative analysis of FR051 (Supplementary Figure 4A-C) and quantitative analysis of FR054 and FR053 (Supplementary Figure 4C) showed that the intracellular concentration of the sum of the three forms and more specifically of FR051 was 925 ± 106 nM and 106 ± 50 nM, respectively, indicating that FR051 has an effect at nM concentration despite its use at higher amount. This result is supported also by an NMR analysis of the extracellular FR054 chemical stability performed by using a ^13^C-labeled FR054. In fact this analysis revealed that upon 24 h, almost 64% of the compound was hydrolyzed also in absence of the cells, and that only around 11% was taken up by cells (data not shown). Therefore such an intrinsic instability may justify the high concentration used in our experiments. Decreased viability of breast cancer cells following FR054 treatment prompted us to investigate the mechanism of cell death. MDA-MB-231 cells were treated with 1 mM FR054 for 48 h and necrosis/apoptosis was measured by a staining with propidium iodide and Annexin V-FITC. FR054 potently increased apoptosis as compared to necrosis (Fig. 2c) and accordingly stimulated caspase-3 activation and Poly-ADP ribose polymerase (PARP) cleavage (Fig. 2d). These data indicated that FR054 induces apoptotic cell death in the less sensitive MDA-MB-231 cells and suggest an equal mechanism also in the other breast cancer cells.

### FR054-dependent MDA-MB-231 apoptosis is repressed by an HBP salvage pathway inhibitor and enhanced in PGM3 knockdown cells

**Fig. 3:**
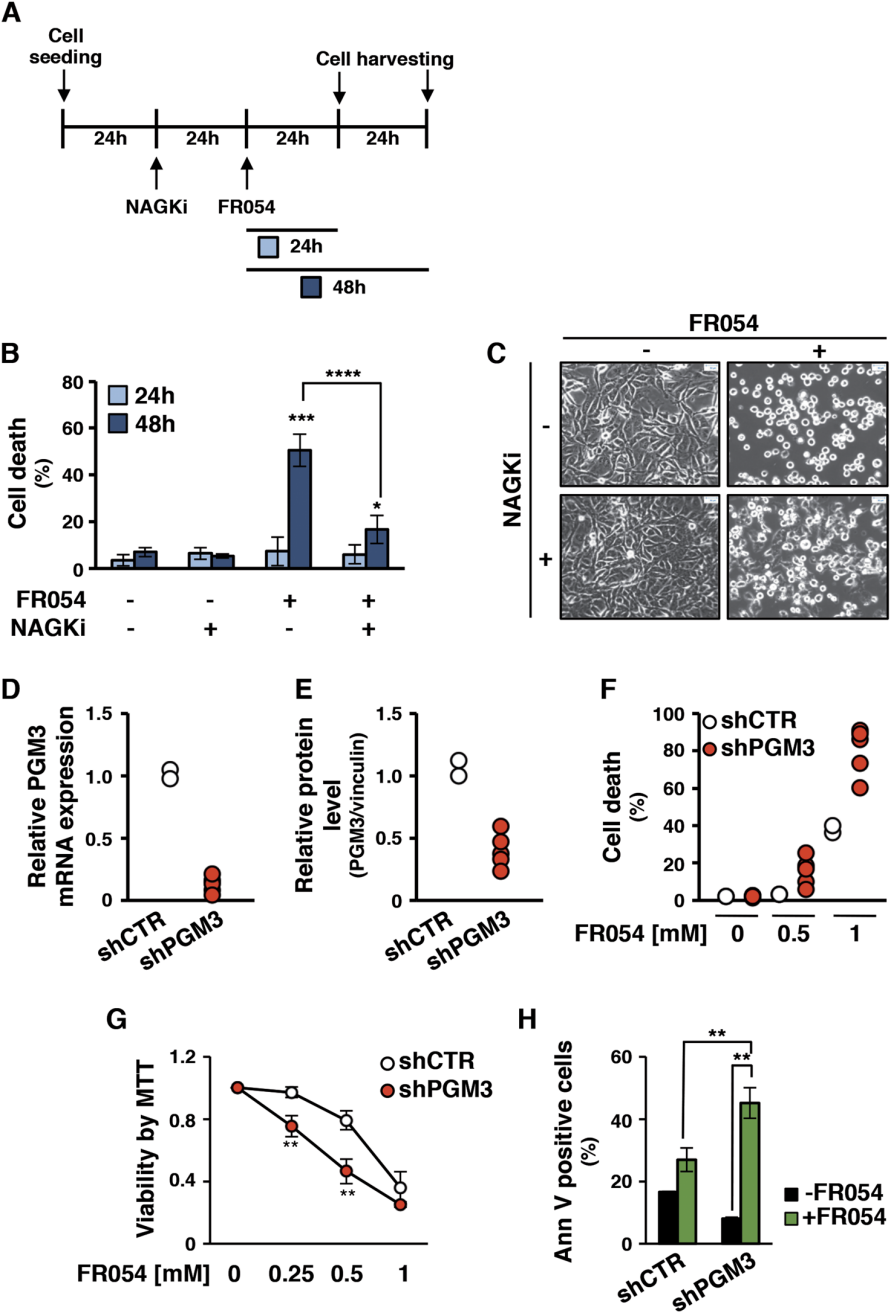
FR054-dependent MDA-MB-231 apoptosis is impaired by a NAGK-specific inhibitor and enhanced in shPGM3 cells. Cell death of MDA-MB-231 cells upon treatment with NAGKi and FR054: scheme of treatment (**a**), cell death determined by viable count (**b**), representative images of cells upon treatment (**c**) (4× magnification, 50 μm scale). Efficacy of shRNA silencing of PGM3 enzyme in MDA-MB-231 stable clones, as detected by RNA (**d**) and protein (**e**) levels quantification. **f** Cell viability of shCTR and shPGM3 clones upon 48 h treatment with FR054 0.5 mM and 1 mM. **g**, **h** Cell viability of MDA-MB-231 shCTR and shPGM3 representative clones detected by MTT test (**g**; 48 h FR054 at different doses) and Ann V-FITC staining (**h**; 48 h FR054 0.5 mM). All data represent the average ± s.d.; *p < 0.05, **p < 0.01 (Student’s *t*-test; comparison with FR054-not-treated sample); *N* = 3

To further confirm the molecular mechanism of the prodrug transformation into the active compound through the HBP salvage pathway, next we evaluated the effect of 3-*O*-methyl-*N*-acetylglucosamine^24^, a competitive inhibitor of the NAGK (named NAGKi), on FR054-induced MDA-MB-231 apoptosis, as shown in Fig. 3a; 24 h pretreatment with NAGKi fairly completely blocked the FR054 cell effect (Fig. 3b, c), confirming the central role of the salvage pathway in its intracellular chemical transformation and activity. Conversely, cell culture supplementation with Glc*N*Ac (ranging from 10 to 50 mM) did not allow cells to bypass cell death induced by FR054 (Supplementary Figure 5A), further confirming the activity of FR054 compound against PGM3 and its action mechanism. To confirm the in vivo effect of FR054 on PGM3, MDA-MB-231 cells expressing an shRNA vector targeting PGM3 or a control sequence were generated. Given that, two different control cell clones (named shCTR) and five PGM3-specific short hairpin cell clones (named shPGM3), characterized by different levels of mRNA and protein (Fig. 3d, e and Supplementary Figure 5B-C), were selected and analyzed upon FR054 treatment. Remarkably shPGM3 clones showed a significant great sensitivity to 48 h FR054 treatment as measured by cell viability at 0.5 and 1 mM FR054 (Fig. 3f and Supplementary Figure 5D). We also analyzed cell viability by MTT assay and cell death by Annexin V in a selected shPGM3 cell clone, namely #3. shPGM3-3 FR054-treated clone showed at 0.25 and 0.5 mM a significant reduced viability and a significant increase of the apoptosis as compared to the control clone (Fig. 3g, h). These results strongly support the conclusion that the effect of FR054 occurs through PGM3 inhibition instead of other off-target effects.

### FR054 treatment efficiently affects both *N*- and *O*-glycosylation levels in MDA-MB-231 cells

**Fig. 4:**
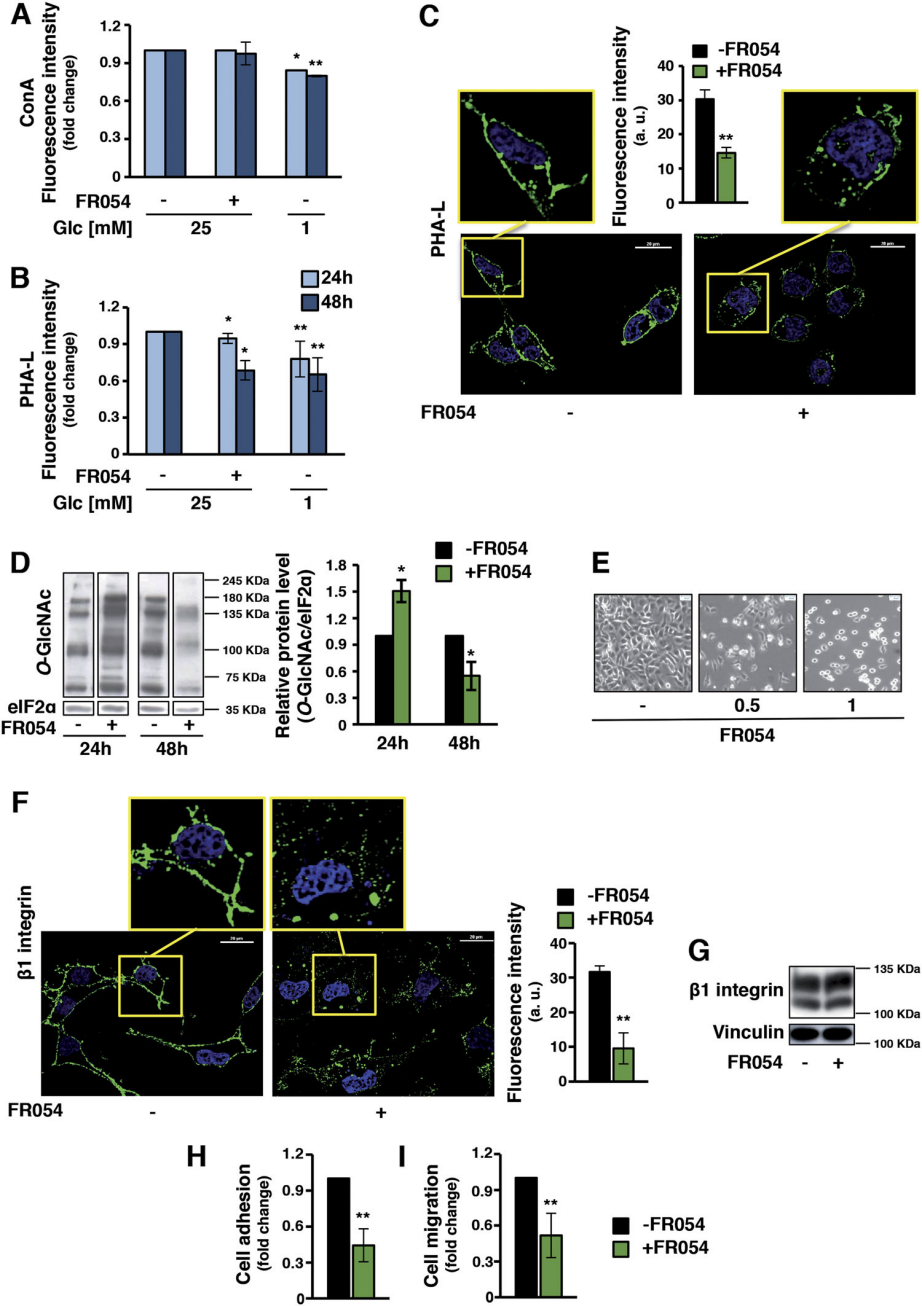
FR054 induces in MDA-MB-231 cells a decrease in *N*-/*O*-Glc*N*Ac protein levels and a reduction of their ability to adhere and migrate. FACS analysis of membrane *N*-glycans in live cells, treated with FR054 or grown in 1 mM glucose, stained with fluorochrome-conjugated ConA (**a**) and PHA-L (**b**). **c** Confocal microscopy of PHA-L staining in MDA-MB-231 cells treated with 250 μM FR054 for 24 h (40× magnification, 20 μm scale) and relative fluorescence intensity quantification. **d** Protein *O*-Glc*N*Ac detection in total cell extract from MDA-MB-231 cells treated with 1 mM FR054 for 24 and 48 h and relative band intensity quantification (right histogram). **e** MDA-MB-231 cells appearance upon 48 h treatment with FR054 (4× magnification, 50 μM scale). **f** Confocal microscopy analysis of β1 active integrin membrane localization in MDA-MB-231 cells treated with 250 μM FR054 for 24 h (40× magnification, 20 μm scale) and relative fluorescence intensity quantification. **g** β1 integrin expression in MDA-MB-231 cells upon treatment with 250 μM FR054 for 24 h. MDA-MB-231 cell adhesion (**h**) and migration (**i**), upon 24 h treatment with 250 μM FR054. All data represent the average ± s.d.; *p < 0.05, **p < 0.01 (Student’s *t*-test; comparison with FR054-not-treated sample); *N* = 3

PGM3 activity reduction in mouse and human models leads to a decrease in both complex branched *N*-glycans and *O*-Glc*N*Ac-modified proteins^12,13^. In fact the membrane expression of tri-/tetra-antennary *N*-glycans in cultured cells as well as protein-*O*-Glc*N*Ac are sensitive to changes in the intracellular amount of UDP-Glc*N*Ac. Since FR054 reduces the UDP-Glc*N*Ac level in the cell extract, next we measured if the reduction could affect intracellular *N*- and *O*-protein Glc*N*Ac. *N*-Glc*N*Ac level was detected by using flow cytometric analysis of specific lectins. Hybrid/high mannose and di-antennary glycans were recognized by *Concanavalin A* (ConA), while tri-/tetra-antennary structures were detected by *Phaseolus vulgaris* (PHA-L). MDA-MB-231-treated cells indicated that the ConA reactivity remained almost similar to untreated ones after both 24 and 48 h (Fig. 4a). Conversely, PHA-L reactivity significantly decreased (Fig. 4b). The latter result was further corroborated by confocal microscopy performed upon 24 h of treatment with no cytotoxic amount of FR054 (250 μM). As shown in Fig. 4c, a 50% decrease of PHA-L membrane reactivity was observed. As control, we used glucose starvation since it has been shown to reduce HBP flux and consequently general lectin reactivity^25^. In fact, both ConA and PHA-L reactivity were significantly reduced under glucose starvation (Fig. 4a, b). *O*-Glc*N*Ac levels were measured by Western blot analysis. After 24 h of treatment, MDA-MB-231 cells showed an increase in *O*-Glc*N*Ac that drastically dropped at 48 h (Fig. 4d), confirming the FR054 affect also on this HBP branch. The results obtained at 24 h were somehow counterintuitive with respect to the inhibition of HBP flux. However, previous reports have indicated that a variety of cellular stressors such as glucose depletion may induce a later increase in *O*-Glc*N*Ac, i.e., by increasing O-linked β-N-acetylglucosamine transferase (OGT) expression^26^. Therefore, we examined the OGT protein expression and accordingly at 24 h we observed around 3-fold induction of OGT protein in FR054-treated samples as compared to control ones, while it was completely lost at 48 h (Supplementary Figure 6A). These data could suggest a specific adaptive mechanism to maintain protein *O*-Glc*N*Ac. To further detail the action of FR054 on HBP as well as on general metabolism, since the strict correlation between HBP and other metabolic pathways^27^, we performed an untargeted mass spectrometry-based metabolomics analysis of 24 h FR054-treated cells as compared to control cells. Pathway enrichment analysis and metabolic map reconstruction, using the metabolites whose changes in abundance discriminated untreated and treated cells (Supplementary Table S1), revealed highly significant enrichment for the pathway targeted by FR054, amino sugar metabolism, and the glutamate/glutathione metabolism interconnected with pantothenate/CoA biosynthesis (Supplementary Figure 6B-C). In fact either HBP metabolites (i.e., Glucosamine-6-P, Glc*N*Ac-6-P + Glc*N*Ac-1-P, UDP-Glc*N*Ac) or glutamate/glutathione/pantothenate/CoA metabolites (i.e., pyridoxal, 4′-phosphopantothenate, cysteine, GSH, GSSG) increased in FR054 samples. This result was in accordance with the increased level of protein *O*-Glc*N*Ac observed after 24 h of treatment delineating an adaptive mechanism for cells, probably to cope with the early inhibitory effect induced by FR054 treatment. However prolonged exposure to FR054 induces cell death in association with a reduction of both highly branched *N*- and *O*-Glc*N*Ac. Moreover, the metabolic analysis pointed out also toward an effect of FR054 on the cell redox system more than a general metabolic reprogramming.

### FR054 reduces MDA-MB-231 cell adhesion, migration, and integrin β1 membrane localization

Changes in membrane protein *N*-Glc*N*Ac, for instance in integrins, may alter cell adhesion and migration^28–31^. Therefore, we tested if FR054 could hamper cell adhesion and migration in MDA-MB-231 cells by interfering with β1 integrin membrane localization. First, we confirmed that FR054 induced a dose-dependent cell detachment not associated to cell death (Fig. 4e and data not shown). Then, we evaluated cell surface-active β1 integrin by confocal microscopy upon 24 h of FR054 (250 μM). Treated cells showed a more clustered surface staining and around 70% reduction of β1 integrin as compared with untreated ones (Fig. 4f). Of note, analysis of β1 protein expression in whole-cell lysates indicated unchanged levels between untreated and treated cells, confirming that FR054 altered β1 membrane localization (Fig. 4g). MDA-MB-231 cell adhesion and migration upon FR054 treatment was also evaluated. FR054-treated cells showed around 60% and 50% reduction in cell adhesion and migration, respectively (Fig. 4h, i). To exclude any possible artifact due to FR054 non-specific interactions with glycosaminoglycans surrounding the cells, PHA-L reactivity, adhesion, and migration were evaluated upon treatment with 1 mM FR053. FR053 did not influence PHA-L reactivity as well as with cell adhesion and migration (Supplementary Figure 7). Altogether these results indicate that FR054 is able to reduce active β1 integrin membrane localization leading to a dramatic reduction of adhesion and migration of MDA-MB-231 cancer cells.

### FR054 induces endoplasmic reticulum (ER) stress and a ROS-dependent apoptotic cell death

**Fig. 5:**
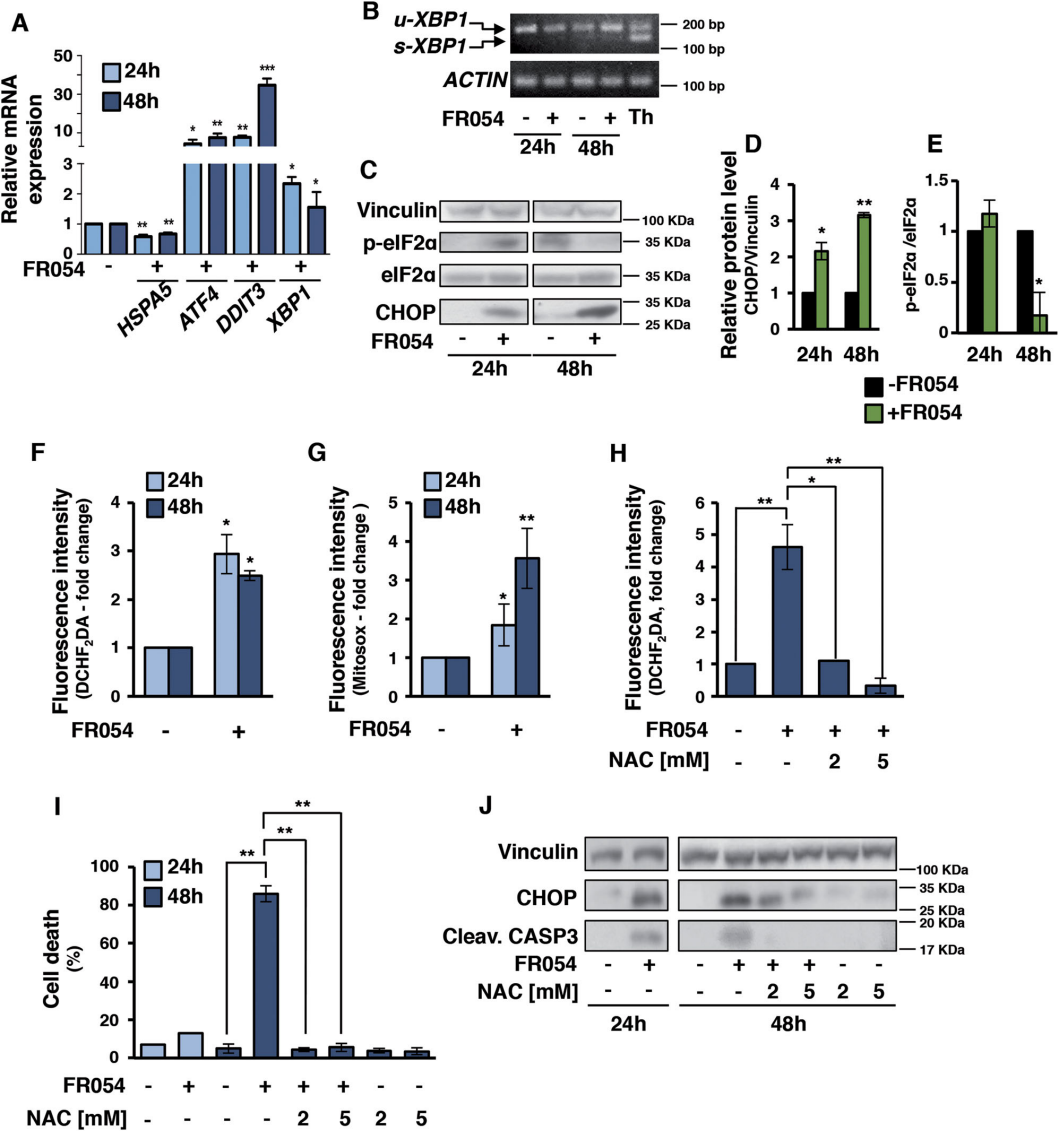
FR054 induces UPR activation and intracellular ROS increase. **a** mRNA expression of *HSPA5*, *ATF4*, *DDIT3*, and *XBP1* in MDA-MB-231 cells following 24 and 48 h of FR054 treatment. **b** Analysis of XBP1 mRNA splicing in MDA-MB-231 cells following 24 and 48 h treatment with FR054 or 6 h with Thapsigargin (Th). u-XBP1 indicates unspliced form and s-XBP1 indicate spliced form. Protein expression (**c**) and densitometric quantification of CHOP (**d**) and eIF2α phosphorylation (**e**) in MDA-MB-231 cells following 24 and 48 h treatment with FR054. Intracellular hydrogen peroxide (**f**) and mitochondrial superoxide (**g**) measured by FACS analysis after DCHF_2_DA and Mitosox staining, respectively, in MDA-MB-231 cells upon treatment with 1 mM FR054 for 24 and 48 h. **h** Hydrogen peroxide levels measured with DCHF_2_DA in MDA-MB-231 upon treatment with 1 mM FR054 for 48 h or co-treated with different doses of NAC. **i** Viable cell count of MDA-MB-231 cells upon treatment with 1 mM FR054 and different doses of NAC. **j** Caspase-3 activation and CHOP expression of the samples described in **i**. All data represent the average ± s.d.; *p < 0.05, **p < 0.01, ***p < 0.001 (Student’s *t*-test; comparison with FR054-not-treated sample); *N* = 3

Among the different functions, *N*-Glc*N*Ac is necessary for protein folding and stability^32^. If *N*-Glc*N*Ac protein level decreases, many proteins with incorrect folding accumulate into the ER leading to severe cellular stress. This condition activates the Unfolded Protein Response (UPR)^33^, leading, if sustained, to cell death. To investigate a possible link between the FR054 effect on cell viability and UPR activation, we evaluated the mRNA and protein expression of some key players of ER stress in MDA-MB-231 cells treated for 24–48 h with 1 mM FR054. As shown in Fig. 5a, mRNA level of *HSPA5*/*GRP78*, an important pro-survival molecular chaperone associated to UPR, appeared to slightly decrease at both time-points. Conversely, *ATF4*, *DDIT3*/*CHOP*, and *XBP1* were significantly upregulated. Of note, the ER-stress-induced splicing of XBP1 (s-XBP1), target of the pro-survival IRE1 branch of UPR, was undetectable in FR054 treated samples as compared to thapsigargin, a classical ER stressor, suggesting a lack of activation of IRE1-dependent XBP1 splicing (Fig. 5b). Protein expression analysis confirmed mRNA data, given that a significant increase of CHOP level was observed (Fig. 5c, d). In agreement with previous reports indicating that apoptosis induced by prolonged ER stress is associated to eIF2α phosphorylation decrease and CHOP increase^34^, also in our experiments p-eIF2α levels decreased at 48 h (Figs. 5c, e). Altogether these results, while confirming that the FR054 is able to induce UPR, as it is predictable for an inhibitor of the HBP, by contrast suggested also a specific effect, since its behavior was partially different from other ER stressors, such as thapsigargin.

Accumulation of unfolded proteins in ER may enhance intracellular ROS levels that may participate in the activation of apoptosis^35^. Since metabolomics data suggested an impact of FR054 on cell redox system, we next assessed whether FR054 was associated with ROS enhancement. FR054 treated cells (48 h) were stained with DCHF_2_DA and MitoSOX for H_2_O_2_ and mitochondrial superoxide detection, respectively. Both types of ROS significantly increased in a time-dependent manner (Fig. 5f, g). Next, ROS role in FR054-dependent cell death was evaluated by testing the ROS antagonist N-acetylcysteine (NAC) (Supplementary Figure 8A). Strikingly, NAC addition, in association with a significant ROS reduction (Fig. 5h), decreased apoptosis (Fig. 5i and Supplementary Figure 8B) and increased cell proliferation (Supplementary Figure 8C). Concurrently, NAC treatment significantly reduced CHOP expression and caspase-3 activation in a dose-dependent manner as compared to FR054 alone (Fig. 5j), supporting an important role of ROS in FR054 action.

### FR054 reduces tumor growth in MDA-MB-231 xenograft mice

**Fig. 6:**
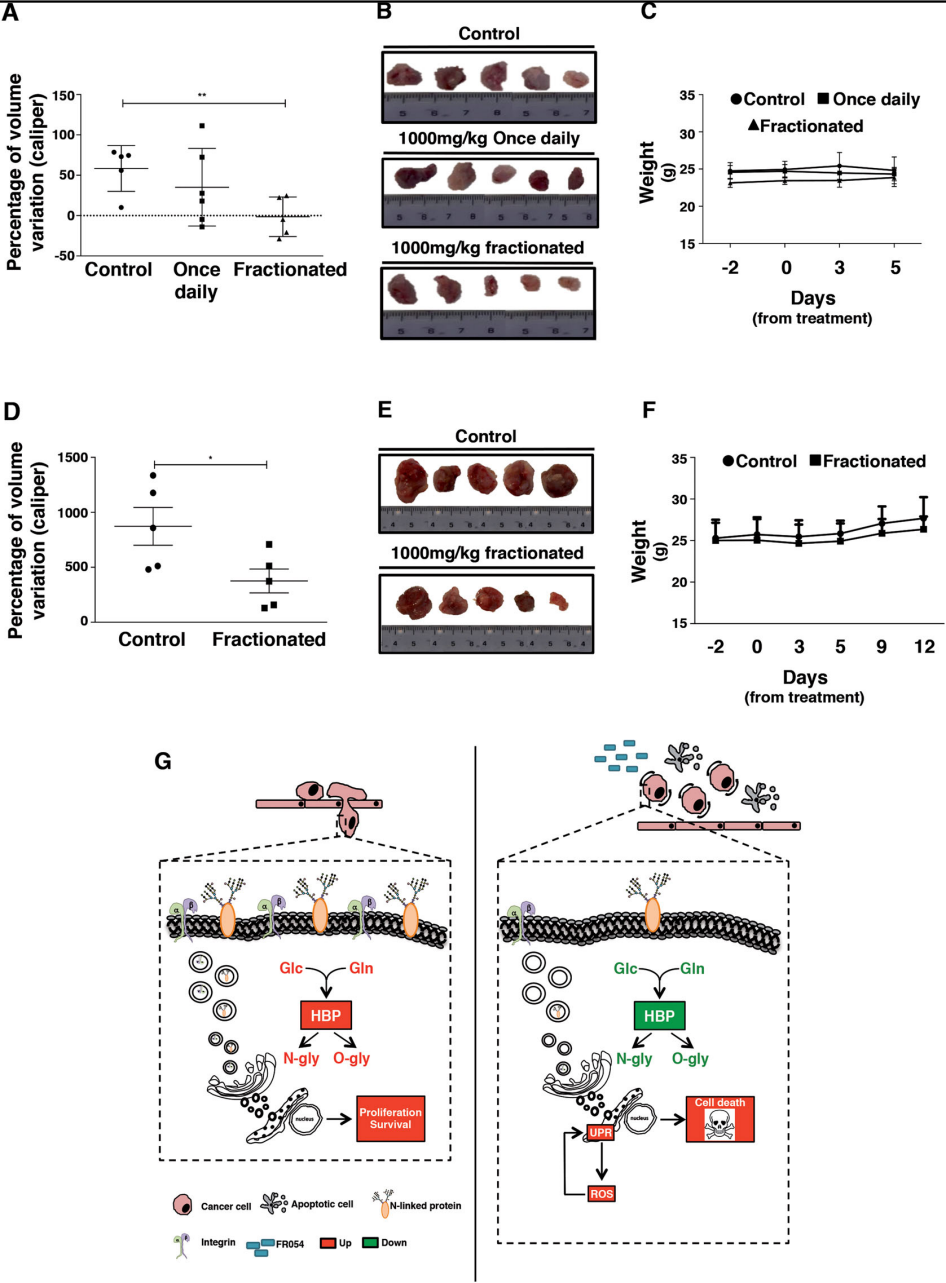
Antitumor effect of FR054 in nude mice xenograft model of TNBC cells MDA-MB-231. **a** Tumor volume variation between pre-treatment and the end of treatment as measured with calliper; **p < 0.01 (Student’s *t*-test); mice/group: *n* = 5 (control, fractionated); *n* = 6 (once daily) upon 5 days of treatment. **b** Representative images of tumor harvested after mice sacrifice at the fifth day of treatment. **c** Mean body weights for each group during 5 days of treatment. **d** Tumor volume variation between pre-treatment and the end of treatment as measured with calliper; *p < 0.05 (Student’s* t*-test); mice/group: *n* = 5 (control, fractionated); upon 11 days of treatment. **e** Representative images of tumor harvested after mice sacrifice at 11th day of treatment. **f** Mean body weights for each group during 11 days of treatment. **g** Schematic representation of the main pathways affected by FR054 treatment in MDA-MB-231 cell line. The significant upregulated mechanisms are depicted in red color while green color is referred to downregulated ones

Animal studies using MDA-MB-231 cells examined the relevance of our observations to tumor growth. Mice were subcutaneously injected with MDA-MB-231 cells; 7 days after cells injection (tumors volume of around 180 ± 40 mm^3^), mice underwent intraperitoneal injection of one concentration of FR054 (1000 mg/kg) administered in single or fractionated dose (twice a day 500 mg/kg/dose) as compared to vehicle alone (10% DMSO). After 5 days of FR054 treatment, mice treated in fractionated dose showed a tumor growth stasis and a tumor volume variation significantly lower than control mice (*p* = 0.008; Fig. 6a, b). Conversely, single dose-treated mice displayed a non-significant difference as compared to control mice (Fig. 6a, b) and a lower tumor growth inhibition index (TGI) as compared to mice treated with fractionated dose (9.69 and 34.62, respectively). No differences in the mice body weights and no apparent signs of morbidity based on body condition scoring were observed (Fig. 6c). On the basis of these results, we investigated the long-term effects of the fractionated treatment (11 consecutive days). The prolonged treatment with FR054 confirmed no toxic effects in mice health conditions, no body weight loss (Fig. 6f) and induced a reduction of tumor growth rate (TGI = 41.77) since the percentage of volume variation was significantly smaller after the prolonged treatment than the control mice (*p* < 0.05) (Fig. 6d). Thus, FR054 appears to have an in vivo antitumor efficacy that is higher when administered twice a day compared to single administration.

## Discussion

High level of *N*- and *O*-protein Glc*N*Ac are associated to several aspects of cancer biology. Given that, different compounds have been identified as possible inhibitors of both or mostly one of the two protein modifications. However, while this approach has been quite successful in vitro, minor results have been obtained in vivo^9,10^.

In the present study, we describe the computational identification, the synthesis, and the evaluation of a novel first-in-class small cell-permeable inhibitor of HBP, FR054. In particular, we demonstrate that FR054 acting as a competitive inhibitor for PGM3 enzyme is able to induce an early decrease of intracellular UDP-Glc*N*Ac, and hence to diminish both HBP branches as confirmed by the reduction of highly branched *N*-glycans and protein *O*-Glc*N*Ac. Former effect is extremely interesting since different oncogenic proteins, such as RTKs, integrins, etc., need this type of highly branched structures for their cell surface retention and signaling. In fact, *Mgat5* knockout mice, the enzyme responsible for the addition of complex *N*-glycan branches, exhibit delayed tumor growth and a general decrease of growth factor signaling^36^. Consistent with these observations, our study revealed that FR054 induces cell proliferation arrest and apoptosis in a heterogeneous set of breast cancer cells, as well as a strong reduction of the metastatic MDA-MB-231 cells to adhere and migrate, suggesting that FR054 can impact a broad range of cancer specific deregulated pathways (Fig. 6d). In accordance, global *N*-Glc*N*Ac inhibition by Tunicamycin (Tun) exerts similar effect on different cancer cells^37,38^. Important confirmation of FR054 role as HBP inhibitor is also provided by its ability to induce ER stress and UPR, as previously observed for the less specific HBP inhibitors Aza and DON^39^ and for Tun^40^. FR054 mechanism of action is also strengthened by its ability to increase intracellular ROS. In fact, prolonged ER stress has been associated also to oxidative stress and cell death^41^. However, FR054 appears to have different advantages as compared to other HBP and *N*-Glc*N*Ac inhibitors. It is able to inhibit both HBP branches, equally important for cancer growth^4^, it is able to avoid cancer cell ability to refuel HBP through the salvage pathway under harsh nutrient tumor condition^25,42^ and since it is not being a glutamine structural analog, is less unspecific and probably less toxic. In fact, it exhibits encouraging efficiency in the treatment in vivo of MDA-MB-231 xenografts mice with no noticeable toxicity, at least in the time frame of our treatment, as well as in diverse human breast cancer cells and pancreatic cancer cells (unpublished results) and minor toxicity in normal cells. Further work will be required to increase FR054 chemical stability that will help to enhance its apparent potency, as we performed the experiments in the high μM to mM range, despite its intracellular concentration that was in the nM range. Nevertheless, our study provides clear indications that PGM3 and in general HBP is a promising target in clinical treatment of tumors that, upon metabolic reprogramming, rely more heavily on the HBP. As a whole, these results merit potential avenues of research for the use of FR054 as a mono- or combinatorial therapy by targeting, for instance, also ER and redox system in cancer cells.

## Materials and methods

### Cell lines and shPGM3 clones

Human breast cancer MDA-MB-231 cells were routinely cultured in Dulbecco’s modified Eagle’s (DMEM) medium containing 25 mM glucose, supplemented with 4 mM L-glutamine, 100 U/mL penicillin, 100 mg/mL streptomycin (complete medium), and 5% fetal bovine serum. Other breast cancer cell lines (SKBR-3, BT-474, Mcf7, T-47D, MDA-MB-361) and Retinal Pigmented Epithelial (hTERT-RPE-1) cells were cultured in the same medium, with exception for serum that was 10%, while MDA-MB-468 were cultured in DMEM containing 25 mM glucose, supplemented with 2 mM L-glutamine, 100 U/mL penicillin, 100 mg/mL streptomycin, and 10% fetal bovine serum. Cells were grown and maintained according to standard cell culture protocols and kept at 37 °C with 5% CO_2_. The medium was replaced every 2–3 days and cells were splitted or seeded for experiment when reached the sub-confluence. All reagents for media were purchased from Thermo Fisher Scientific (Waltham, MA, USA).

Silencing of PGM3 was obtained transfecting MDA-MB-231 cells with plasmid from Sigma-Aldrich (St. Louis, MO, USA) (MISSION shRNA for PGM3: NM_015599, clone ID: NM_01559.1-1309s21c1); also control plasmid (shCTR, not human or mouse shRNA) was purchased from Sigma-Aldrich (SHC002). To transfect cells Polyfect transfection reagent (QIAGEN, Hilden, Germany) was used. Briefly, 2 × 10^5^ cells were seeded in MW6 well, after 24 h 1.5 μg DNA was added to 100 μL of medium (opti-mem, Thermo Fisher Scientific) and mixed; after that 10 μL of transfection reagent was added to the mix, vortexed and incubated at room temperature for 10 min; then 0.6 mL of cell medium was added and the transfection mixture distributed in the cell well containing 1 mL of complete cell medium. To select clones presenting stable transfection, cells were treated with 2 μg/mL puromycin (Euroclone, Pero, Italy) for a week and then seeded in MW96 wells at a density of 1 cell/well under puromycin selection. After their proliferation and expansion, different clones were tested for PGM3 levels.

### Cell treatment and cell viability evaluation

Where not differently specified, for experiments, cells were seeded in complete growth medium and after 24 h washed twice with phosphate buffer saline (PBS) and incubated in complete medium with or without FR054 or FR053. In some cases, a double treatment was performed and the molecules (FR054 with Glc*N*ac, NAC or NAGKi) added to the cells as indicated in specific experimental schemes presented in the text or figures. As control of UPR activation, the cells were treated with Thapsigargin.

All chemicals and inhibitors were purchased from Sigma-Aldrich, except for Thapsigargin (Vinci-Biochem, Firenze, Italy) and NAGKi (3-O-methyl-N-acetyl-D-glucosamine; Cayman, purchased from Vinci-Biochem).

To measure cell proliferation, harvested cells were counted using the Burker chamber. Where indicated, cell viable count was performed using Trypan Blue Stain 0.4% (Thermo Fisher Scientific).

Cell viability was measured also with MTT test (Roche from Sigma-Aldrich) according to the manufacturer’s protocol. Briefly, 5 × 10^3^ cells were seeded into 100 μL of medium in 96 flat bottom multiwell. At a specific time point, 10 μL of the labeling reagent was added to the cells. After 4 h at 37 °C in humidified atmosphere, 100 μL of the solubilization solution was added, and the cells incubated overnight. After that, the absorbance at 620 nm was measured with an ELISA reader; absorbance at 800 nm was used as reference.

For the colony formation assay, after specific treatment, the cells were collected and then re-seeded at low density in complete medium. After 12 days, cells were washed twice with PBS, fixed in PBS-formaldehyde 5%, and stained with 0.1% crystal violet for 5 min. After colorant dissolving by 10% acetic acid, the absorbance was analyzed using a spectrophotometer.

### Chemical synthesis and computational analysis

All solvents were dried with molecular sieves for at least 24 h prior to use. Thin-layer chromatography (TLC) was performed on silica gel 60 F254 plates (Merck, Kenilworth, NJ, USA) with detection using UV light when possible, or by charring with a solution of concd. H_2_SO_4_/EtOH/H_2_O (5:45:45) or a solution of (NH_4_)_6_Mo_7_O_24_ (21 g), Ce(SO_4_)_2_ (1 g), concd. H_2_SO_4_ (31 mL) in water (500 mL). Flash column chromatography was performed on silica gel 230–400 mesh (Merck). ^1^H NMR spectra were recorded at 25 °C, with a Varian Mercury 400 MHz instrument, and processed with MestReNova v6.0.2–5475 software. Chemical shift assignments, reported in ppm, are referenced to the corresponding solvent peaks (omitted in the peak assignment). MS were recorded on a QSTAR elite LC/MS/MS system with a nanospray ion source (Agilent).

Compounds FR053 and FR054 were synthesized according to the procedure described in ref.^20^ and the spectroscopic characterization is in accordance to the reported data.

FR054: 2-methyl-(3,4,6-tri-*O*-acetyl-1,2-dideoxy-α-d-glucopyrano)-[2,1-d]-2-oxazoline. Briefly, 2-acetamido-1,3,4,6-tetra-*O*-acetyl-2-deoxy-α-d-glucopyranose (1.57 g, 4.03 mmol) was refluxed for 18 h in dry CH_2_Cl_2_ (20 mL) under argon in the presence of TMSOTf (1.09 mL, 6.04 mmol, 1.5 equiv.). The reaction mixture was then quenched with Et_3_N (2 mL), evaporated under reduced pressure, and the residue was purified by flash chromatography over silica gel (eluent: Petroleum Ether: AcOEt = 1:9) to yield 1.26 g (3.82 mmol, 95%) of FR054 as a pale brown oil.

FR053: 2-methyl-(1,2-dideoxy-α-d-glucopyrano)-[2,1-d]-2-oxazoline. To a solution of compound FR054 (200 mg, 607 mmol) in anhydrous MeOH (10 mL), a catalytic amount of sodium methoxide (16.2 mg, 300 mmol, 0.5 equiv.) was added. The reaction was stirred at room temperature for 2 h, then quenched with resin IR-120 H. After filtration, the solvent was removed under reduced pressure, and the crude product FR053 was used without further purification.

All molecular docking calculations were performed using the PGM3 crystal structure from *Candida albicans* (Protein Data bank code: 2dkc) co-crystallized with the natural substrate (GlcNAc-6-P). The sequence identity over the entire protein between human PGM3 (Hs-PGM3) and PGM3 of *Candida albicans* (Ca-PGM3) is 48%.

The docking scores were computed with the software Schrodinger 10.1 Maestro and the docking calculations were performed using the Glide docking module^43^, considering a protonation state compatible with pH = 7, and sampling a box (18 × 18 × 18 Å^3^) centered on the enzyme active site. All ligands were docked with the extra precision (XP) method and explicitly taking into account the conformational flexibility of ligands.

In order to obtain the lowest conformational energy, the structures of the protein and the ligand (substrate or new molecules) were first prepared (addition of hydrogens atoms, assignment of atomic charges and bond orders, elimination of water molecules not involved in ligand binding) and optimized within the Protein Preparation Wizard, using the force field OPLS_2005.

### Cellular thermal shift assay (CETSA)

The ability of compounds to interact with and thereby stabilize the target in intact cells was analyzed essentially as previously described^44^. Briefly, cells cultured in 100 × 20 mm tissue culture dishes at 90% confluence were collected with PBS supplemented with protease inhibitor cocktail (Sigma-Aldrich) and phosphatase inhibitors (Sigma-Aldrich). Cells were freeze–thawed three times using liquid nitrogen and centrifuged at 16,000*g* for 30 min, thus protein soluble fractions were transferred to new tubes at 4 °C and distributed in aliquotes into PCR tubes and incubated with FR054 or vehicle for 30 min RT. After incubation, PCR tubes were heated for 3 min from 49 to 70 °C followed by cooling for 3 min at room temperature. Precipitated proteins were separated from the soluble fraction by centrifugation at 16,000*g* for 30 min. Soluble proteins, collected in the supernatant, were kept at 4 °C until Western blot analysis. Equal amounts of proteins were loaded onto 10% SDS–PAGE gels, transferred to nitrocellulose membranes, and analyzed using the following antibodies: PGM3 (#A304-555A, Bethyl Laboratories, Montgomery, TX, USA; 1:5000), vinculin (#sc-5573, Santa Cruz Biotechnology Inc., Santa Cruz, CA, USA; 1:10000), UAP1 (HPA014659, Sigma-Aldrich; 1:250). Protein expression levels on Western blots were quantified by densitometry analyses using the ImageJ. The same procedure was performed also at a specific temperature (58 °C) with different concentrations of FR054 and Glc*N*Ac-6-P (Sigma-Aldrich), in the assay called isothermal dose-response fingerprint (ITDRF_CETSA_)^44^.

### UDP-Glc*N*Ac quantification by HPLC

To assess the inhibitory potential of FR054, UDP-Glc*N*Ac, the final product of HBP, was quantified after an in vitro assay exploiting coupled enzymatic reactions. Protein extracts from MDA-MB-231 were obtained disrupting cells with a specific buffer containing 200 mM Tris-HCl pH 7.5, 150 mM NaCl, 1% (v/v) NP-40; 0.1% (w/v) SDS, protease inhibitor cocktail (Sigma-Aldrich) and phosphatase inhibitors (Sigma-Aldrich); 150 µg of protein extract were used for the enzymatic assay with the other components of the reaction: 50 mM Tris-HCl pH 7.5, 5 mM MgCl2, 5 mM Glc*N*Ac-6-P, 5 mM UTP, 3.6 µM glucose-1,6-bisphosphate and 1 mM FR054. After 30 min at 37 °C, UDP-GlcNAc was separated and quantified by high performance liquid chromatography (HPLC; Agilent, Santa Clara, CA, USA), following the method described in^45^ with some modifications and using the column with reverse phase LiChroCART 250-4 LiChrospher 100 RP-18 (5 μm) (Merck) and UV/Vis detector set to detect nucleotide sugars at 254 nm. The elution in the column was performed using a gradient method, with buffer A (100 mM potassium phosphate buffer pH 6.4 supplemented with 8 mM tetrabutylammonium hydrogensulphate) and buffer B (70% 100 mM potassium phosphate buffer pH 6.4 with 30% acetonitrile). The applied elution gradient was the following: 100% buffer A for 15 min; 0–77% buffer B for 20 min; 77–100% buffer B for 1 min; 100% buffer B for 10 min. The flow rate was maintained at 0.8 mL/min. The UDP-Glc*N*Ac peak was identified by comparison with the retention time of a standard injected before the analysis and quantified by integration based on the peak areas of the calibration curve. All chemicals used in this method were purchased from Sigma-Aldrich.

### Flow cytometric analysis

All flow cytometric analysis were performed using a FACScan flow cytometer (Becton-Dickinson, Franklin Lakes, NJ, USA) with CellQuest software (Becton-Dickinson). Propidium iodide (PI)/Annexin V-FITC staining was performed using Apoptosis assay kit from Immunological Sciences (Rome, Italy). In particular, 5 × 10^5^ cells were collected in 50 μL of binding buffer and stained with 1 μL of Annexin V-FITC and 1 μL of PI, for 15 min at room temperature. After the incubation, samples were diluted in an appropriate volume of binding buffer (10 mM HEPES/NaOH pH 7.4, 140 mM NaCl, 2.5 mM CaCl_2_) and analyzed.

Intracellular and mitochondrial ROS levels were measured by staining cells with 5 μM dichloro-dihydro-fluoresceine-diacetate (DCHF_2_DA, Thermo Fisher Scientific) and 5 μM Mitosox (Thermo Fisher Scientific), respectively, for 30 min at 37 °C in medium without phenol red. After staining, the cells were trypsinized, collected in PBS plus serum and analyzed.

To determinate cell surface expression of *N*-linked glycoproteins, after collection 5 × 10^5^ cells were stained with 120 μg/mL ConA, Alexa Fluor 594 conjugate lectin, or 5 μg/mL *P. vulgaris* (PHA-L), Alexa Fluor 488 conjugate lectin, diluted in a specific buffer (10 mM HEPES/NaOH pH 7.4, 140 mM NaCl, 2.5 mM CaCl_2_) for 1 h on ice and then analyzed. Both lectins were purchased from Thermo Fisher Scientific.

### Confocal fluorescence microscopy

1 × 10^5^ cells/well were seeded onto clean glass slides (Knittel glass purchased from VWR International, Radnor, PA, USA) lodged in 6-well plates and incubated for 24 h under normal growth conditions treated or not with 250 μM FR054. To evaluate alterations in β1 integrin organization, cells were washed with complete PBS (containing Ca^++^ and Mg^++^) and incubated with anti-β1 integrin antibody (#AB1952, Millipore, Burlington, MA, USA) diluted in a buffer modified to preserve membrane integrity (10 mM HEPES/NaOH pH 7.4, 140 mM NaCl, 2.5 mM CaCl_2_, 1.8 mM MgCl_2_, 1% BSA and 10% goat serum) for 1 h at 37 °C. Then the cells were washed twice with 1% BSA in complete PBS, followed by incubation with Alexa Flour 488 conjugated anti-mouse (Thermo Fisher Scientific) for 30 min at room temperature in the dark. For detection of cell surface expression of N-linked glycoproteins, cells were stained with 5 μg/mL PHA-L for 1 h. In both cases after washing, the coverslips were fixed with 1% paraformaldehyde for 10 min RT, incubated with DAPI (4′,6-diamidino-2-phenylindole) 2 μg/mL for 10 min at room temperature and mounted with dabco anti-fading reagent (Sigma-Aldrich). Cells were examined under A1R Nikon (Nikon, Tokyo, Japan) laser scanning fluorescence confocal microscope at a magnification of 40× to obtain a minimum of 5 frames per field. Fluorescence emission was quantified using NIS-Elements AR analysis 4.10.00 software by Nikon. To measure the fluorescence of the region of interest, centered on the cell membrane, each cell in the field was considered as a separate event whose intensity florescence is put in mean with all events captured for the sample and reported in graph. Data presented are the means of results from five independent experiments performed in duplicate with a minimum of 15 spots per sample observed.

### Adhesion and migration assays

For attachment assays, after a treatment of 24 h with 250 μM FR054 or FR053, 1 × 10^5^ cells/sample were washed, resuspended in DMEM-0.5% serum, seeded and allowed to adhere for 1 h at 37 °C in 12-well plates coated overnight at 4 °C with 0.1% heat-denatured BSA. Non-adherent cells were then removed by gentle washing with PBS containing Ca^++^ and Mg^++^ (Euroclone) whereas adherent cells were trypsinized and counted.

Cell migration assays were performed in Boyden chambers (Neuro Probe purchased from Euroclone), as previously described with minor modifications^22^. Briefly, 1 × 10^5^ cells/chamber grown for 24 h with 250 μM FR054 or FR053 were detached by mild trypsinization, resuspended in serum-free DMEM, and inoculated into the upper compartments of Boyden chambers. 1% serum diluted in DMEM with 0.1% bovine serum albumin (BSA) was used as chemoattractant in the lower compartments. The two compartments are defined by 8-μm pore size polyvinylpyrrolidone-free (PVPF) filters (0.33 cm^2^, Sigma-Aldrich) coated overnight at 4 °C with 50 μg/mL collagen VI (Sigma-Aldrich) in order to accelerate cell attachment. After 4 h of incubation at 37 °C, the cells on the upper side of the filters were removed by scraping and cells on the lower side of the filters were stained with Mayer’s hematoxylin (Sigma-Aldrich) and counted.

### RNA extraction and semiquantitative RT-PCR analysis

RNA was extracted from cultured cells using Trizol reagent (Thermo Fisher Scientific). Total RNA was reverse-transcribed with oligo dT by using the QuantiTect Reverse Transcription Kit, Qiagen, according to the manufacturer’s protocol; 0.2 μg of the product of reverse transcription was amplified by qPCR with an Applied Biosystem 7500 standard system (Thermo Fisher Scientific) using POWER SYBR GREEN PCR mix for qPCR (Thermo Fisher Scientific). Primers were designed using Primer3Plus software (http://www.bioinformatics.nl/cgi-bin/primer3plus/primer3plus.cgi) and used at 0.25 μM. The relative level of expression was calculated by the 2−ΔΔCT method β-actin was used as endogenous control. Primers are the following: PGM3 forward AGGCAGCTGGTGATGCTATT, reverse GGTCATTGATTGCCTCCTGT; ATF4 forward CCAGACGGTGAACCCAATTG, reverse TCACTGCCCAGCTCTAAACT; DDIT3 forward CCACTCTTGACCCTGCTTCT, reverse TGGTTCTCCCTTGGTCTTCC; HSPA5 forward CGGTCTACTATGAAGCCCGT, reverse CATCTGGGTTTATGCCACGG; XBP1 forward GAGTTAAGACAGCGCTTGGG, reverse GATGTTCTGGAGGGGTGACA; Actin forward GCCTTTATTGCTTCCAGCAG, reverse CGTGGATGCCACAGGACT.

### Western blot analysis

Cells were harvested and disrupted in a buffer containing 50 mM HEPES pH 7.5, 150 mM NaCl, 1% (v/v) glycerol; 1% (w/v) Triton X-100, 1.5 mM MgCl_2_, 5 mM EGTA, protease inhibitor cocktail (Sigma-Aldrich), and phosphatase inhibitors (Sigma-Aldrich); 10–50 μg of total proteins were resolved by SDS-PAGE and transferred to the nitrocellulose membrane, which was incubated overnight with specific antibodies: vinculin (#sc-5573, 1:10000), Eif2α (#sc-133127, 1:1000), CHOP (GADD153, #sc-7351, 1:200), and OGT (#sc-32921, 1:200) from Santa Cruz Biotechnology Inc.; cleaved caspase 3 (#9662, 1:500), PARP (#9532, 1:1000), Phospho- Eif2α (#9721, 1:1000) from Cell Signaling Technology Inc., Danvers, MA, USA; α-*O*-Glc-NAc (Clone RL2, #MA1-072, 1:1000) from Thermo Fisher Scientific; PGM3 (#A304-555A, 1:5000) from Bethyl Laboratories; β1 Integrin (#MAB1951, 1:600) from Millipore. Protein expression levels on Western blots were quantified by densitometry analysis using the ImageJ.

### Mouse xenografts

To generate the mouse model, 5 × 10^6^ MDA-MB-231 (200 µL of DMEM and Matrigel, 1:1 vol/vol) were subcutaneously injected on the right flank of female Athymic nu/nu mice of about 6 weeks of age (approximately 25 g of weight) (Envigo, Bresso, Italy). Animals were kept under specific pathogen-free conditions, handled and maintained according to San Raffaele Institutional Animal Care and Use Committee (IACUC) ethical regulations and the national low. Prior to the beginning of the study, the experimental protocol was approved by the Italian Minister of Health. After cells injection, mice were monitored for body weight and tumor growth. This last was measured using a digital caliper and volumes calculated according to the following formula: tumor volume = [long side×(short side)^2^)/2]. The FR054 was dissolved in H_2_O and 10% DMSO and the dose of 1000 mg/kg was administered with intraperitoneal (i.p.) injection as a single or fractionated dose. When tumor volume reached 180 mm^2^, mice were randomly divided in three groups (5 or 6/group) and treated with vehicle or FR054 once a day (at 9 a.m.) with a dose of 1000 mg/kg (control and once daily groups, respectively) or twice a day (at 9 a.m. and 17 p.m.) with 500 mg/kg of FR054 (1000 mg/kg total as daily dose, fractionated group) for 5 days. Mice weight and tumor volume were daily monitored as previously described. TGI was calculated using the following formula: TGI = [1-(*Tf*/*T*0)*A*/(*Tf*/*T*0)*V*×100], where *Tf* is the last time point analyzed, *T*0 is the initial time point, *A* is the treated group, and *V* is the vehicle^46^. At the end of the studies, mice were sacrificed by CO_2_ asphyxiation and tumors collected for post-mortem analysis.

### Mass spectrometry to detect intracellular molecules

#### Sample preparation

About 7 × 10^6^ MDA-MB-231 cells (untreated and treated with FR054) were collected after trypsinization and washed twice in PBS. Cell pellet was collected in 1.5 mL tubes and kept in ice. The dried pellet was resuspended using 70 μL of solution D. The analyte calibrants (Supplementary Table S1) were treated with the same procedure employed for the sample. The calibration of the compounds FR051 (de-acetylated and phosphorylated form of FR054) was obtained using a multiple chemiometric approach due to the lack of the analytical standard. The quantification data of this analyte are semiquantitative. The MRM transition 282− >97 has been selected to quantify the compounds. The selectivity of the MRM transition has been evaluated on the basis of a qualifier signal at *m*/*z* 242.9 and its structure has been defined through fragmentography tandem mass spectrometry (MS/MS) studies.

#### Mass spectrometry

Acquisition parameters, unless stated otherwise, were as follows. The direct infusion flow rate, provided by means of a syringe pump, was 5 μL/min with additional solvent provided at a flow rate of 100 μL/min by a HPLC pump placed in-line. A Bruker Daltonics HCTultra Ion-Trap mass spectrometer (Bruker Daltonics, Breme, Germany) was used. The spectra were obtained in the positive and negative mode. An average of two microscans and a rolling average of four spectra were acquired. The nitrogen drying-gas temperature was 350 °C, and its flow rate was 1 L/min. The nitrogen nebulizing-gas flow rate was 12 L/min. The internal capillary voltage was between –100 and −1500 V. The end-plate potential was –500 V. The N_2_ curtain gas flow rate was varied between 0.5 and 6 L/min. The ionization sources parameters were fixed as follows: ESI ionization voltage (3000 V) was applied via a home-built external power supply to the spray needle, the APCI needle current was 3000 nA, and the SACI surface potential was 47 V used to obtain the in source ionization and the inter capillary voltage gradient was employed to produce the CIMS effect. HyStar software (Bruker Daltonics, Breme, Germany) was used for data acquisition. DataAnalysis (Bruker Daltonics) was used for data treatment.

### Metabolomics

#### Untargeted metabolomics approach (FIA-Orbitrap-MS/MS)

Metabolites were extracted as previously reported in ref.^47^. The analyses were performed by Flow-Injection Analysis (FIA) using an Agilent 1200 Series coupled to an LTQ-Orbitrap XL mass spectrometer (Thermo Fisher Scientific) equipped with an electrospray source operated in negative and positive mode. Each direct introduction analysis was carried out by injecting 8 μL of sample extract at a flow rate of 50 μL/min of mobile phase consisting of isopropanol/water (60:40, v/v) buffered with 5 mM ammonium at pH 9 for negative mode and methanol/water (60:40, v/v) with 0.1% formic acid at pH 3 for positive mode. Reference masses for internal calibration were used in continuous infusion during the analysis (*m*/*z* 210.1285 for positive and *m*/*z* 212.0750 for negative ionization). Mass spectra were recorded from *m*/*z* 50 to 1000 with 60,000 resolutions. Source temperature was set to 240 °C with 25 L/min drying gas and a nebulizer pressure of 35 psig. MS/MS fragmentation pattern of the significant features was collected and used to confirm metabolite identity. Before each sample, a blank sample (isopropanol/water (60:40, v/v) negative, methanol/water (60:40, v/v) with 0.1% formic acid positive) was run to minimize the carry-over effect. This method allows a rapid metabolic profiling of polar and non-polar compounds. Lipids were considered only by classes due to the intrinsic method limitation in the discrimination of isobaric forms.

All steps of data processing and analysis were performed with Matlab R2016a (The Mathworks, Natick) using in-house developed script following the workflow proposed by^48^. Centroid *m*/*z* lists were exported to csv format. Briefly, in this procedure, we first subtract from each sample its relative blank sample to minimized the carry-over effect then, we applied a cutoff to filter peaks of less than 300 ion counts for negative and 500 ion counts for positive ionization to avoid detection of features that are too low to be statistically significant. Centroid *m*/*z* lists from different samples were merged to a single matrix by binning the accurate centroid masses within the tolerance given by the instrument resolution (about 6 ppm). The output *m* × *n* matrix contains the *m* peak intensities of each mass for the *n* analyzed samples. Because mass axis calibration is applied online during acquisition, no *m*/*z* correction was applied during processing to correct for potential drifts.

Output *m*/*z* list was submitted to statistical analysis (univariate pairwise comparison Mann–Whitney–Wilcoxon Test, JMP pro12, SAS) in order to select features with a statistical significance between FR054-treated and untreated groups of comparisons. Significant altered features were identified by database searches (HMBD, METLIN, http://www.hmdb.ca/, http://metlin.scripps.edu) in positive and negative ionization considering protonate/deprotonate ion associated with Na or K adducts. Bold metabolite refers to metabolites with accurate mass match <6 ppm and an MS/MS fragmentation patterns similarity >99% relative to reference compound present in the database.

#### Metabolic pathway analysis

For biological interpretation of the metabolite dataset, we mapped the significant metabolites (Mann–Whitney–Wilcoxon Test, *p* < 0.05) to the KEGG pathway database (Kyoto Encyclopedia of Genes and Genomes; (www.genome.jp/kegg/), using MetaboAnalyst 3.0, a comprehensive online tool suite for metabolomic data analysis and interpretation (www.metaboanalyst.ca). Enrichment analysis (EA) tools were used to identify metabolic pathways that were most likely to be associated with FR054 treatment.

### Statistics

Unless otherwise noted, all results shown as averages are presented as mean ± s.d. from three or more independent experiments and statistical significance (*p < 0.5, **p < 0.01, ***p < 0.001, ****p < 0.0001) was determined using Student’s *t*-test.

## Electronic supplementary material

Supplemental material
