# Supplementary material for: Inhibition of the Hexosamine Biosynthetic Pathway by targeting PGM3 causes breast cancer growth arrest and apoptosis
Source: Cell Death Dis. 2018 Mar 7;9(3):377. doi: 10.1038/s41419-018-0405-4 (PMC5841296; doi:10.1038/s41419-018-0405-4)
Supplement: Supplementary file 1 — Supplemental material [file 41419_2018_405_MOESM1_ESM.pdf]

Supplementary Material:

Inhibition of the Hexosamine Biosynthetic Pathway by targeting PGM3 causes breast cancer growth arrest and apoptosis

Francesca Ricciardiello[1], Giuseppina Votta[1], Roberta Palorini[1], Isabella Raccagni[2], Laura Brunelli[3], Alice Paiotta[1], Francesca Tinelli[1], Giuseppe D'Orazio[1], Silvia Valtorta[2], Luca De Gioia[1], Roberta Pastorelli[3], Rosa Maria Moresco[2], Barbara La Ferla[1], Ferdinando Chiaradonna[1]*

[1]Department of Biotechnology and Biosciences, University of Milano-Bicocca, Milan, 20126, Italy

[2]Experimental Imaging Center, IRCCS San Raffaele Scientific Institute, Milan, 20132, Italy

[3]Environmental Health Sciences Department, Istituto di Ricerche Farmacologiche Mario Negri, Milan, 20156, Italy.

* Corresponding author :

Ferdinando Chiaradonna, Department of Biotechnology and Biosciences, University of Milano-Bicocca, 2016 Milan, Italy. Phone: +39 02 64483526. E-mail: ferdinando.chiaradonna@unimib.it

Running Title: Breast cancer growth and Hexosamine Pathway

A

FR054: 2-methyl-(3,4,6-tri- *O*-acetyl-1,2-dideoxy-α-d-glucopyrano)-[2,1- d]-2-oxazoline

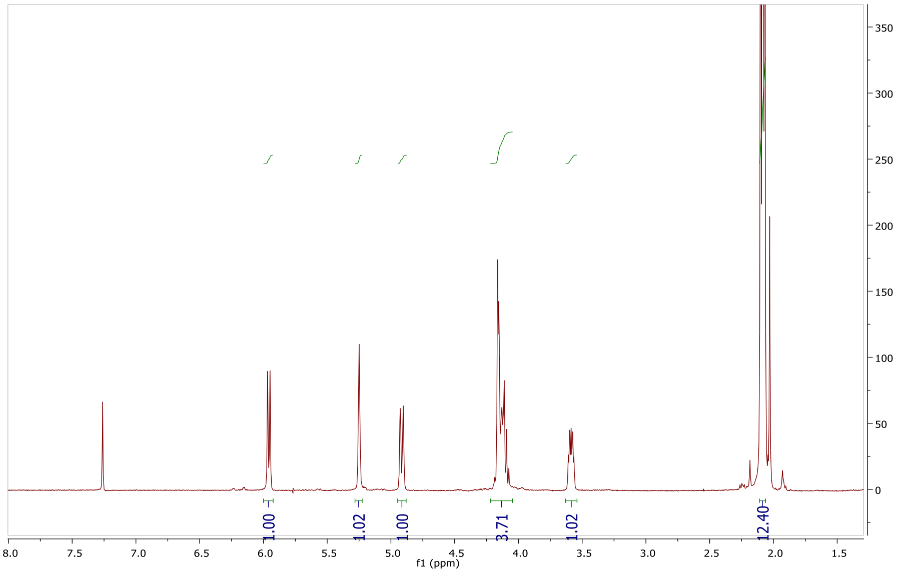

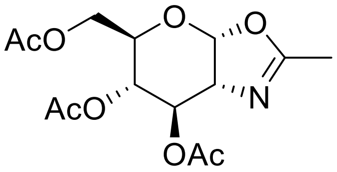

^{1}H NMR (400 MHz, $CDCl_3$) δ 5.96 (d, *J* = 7.4 Hz, 1H), 5.25 (s, 1H), 4.92 (d, *J* = 9.3 Hz, 1H), 4.20 – 4.07 (m, 4H), 3.63 – 3.54 (m, 1H), 2.23 – 1.98 (m, 12H).

C

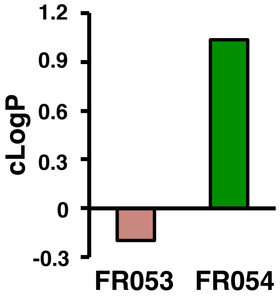

B

FR053: 2-methyl-(1,2-dideoxy-α-D-glucopyrano)-[2,1- d]-2-oxazoline

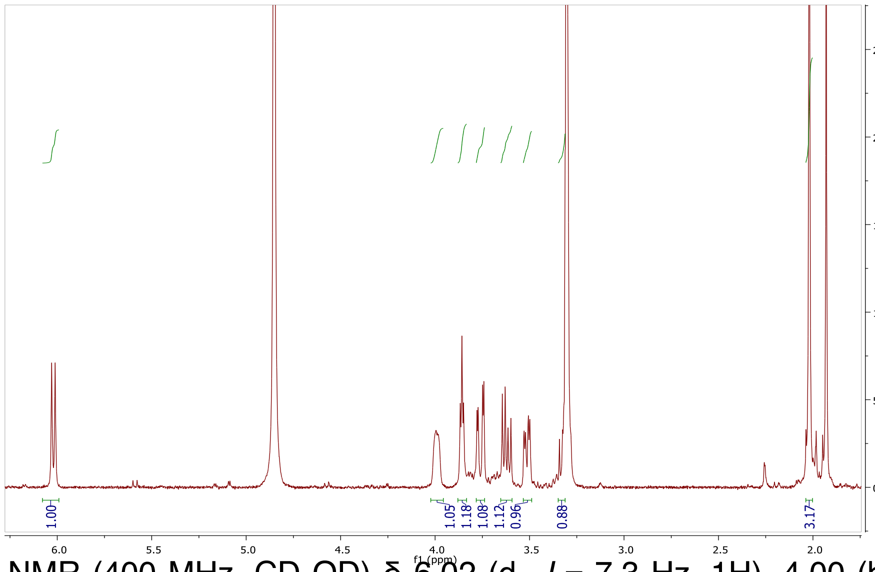

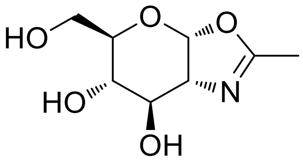

^{1}H NMR (400 MHz, CD_3OD) δ 6.02 (d, *J* = 7.3 Hz, 1H), 4.00 (bs, 1H), 3.86 (t, *J* = 3.5 Hz, 1H), 3.76 (dd, *J* = 12.0, 2.4 Hz, 1H), 3.62 (dd, *J* = 12.0, 6.1 Hz, 1H), 3.51 (dd, *J* = 9.1, 3.1 Hz, 1H),3.35-3.31 (m, 1H), 2.02 (s, 3H).

Supplementary Figure 1. Nuclear Magnetic Resonance (NMR) of FR054 and FR053 chemical structures.
Compound FR054 was synthetised according to the procedure described by Haddoub et *al*. (1) and the spectroscopic characterization is in accordance to the reported data. NMR spectra of (A) FR054 and (B) FR053 and (C) cLogP values of FR053 and FR054 are shown.

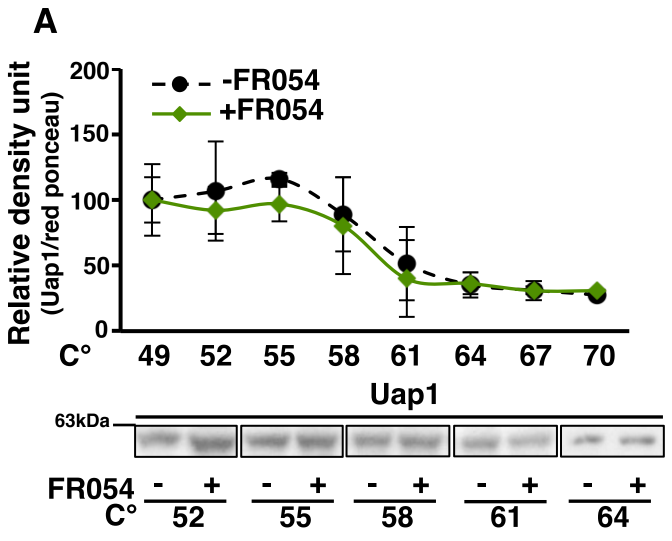

Supplementary Figure 2, related to Figure 1. CETSA melting curves reveal that FR054 *in vitro* treatment does not affect Uap1 and Vinculin protein stability.

CETSA curves in cell lysates for (A) Uap1 and (B) Vinculin protein with FR054 from 49°C to 70°C.

All data represent the average ± s.d., no significance (Student's t-test; comparison +FR054 *vs.* –FR054); N=3.

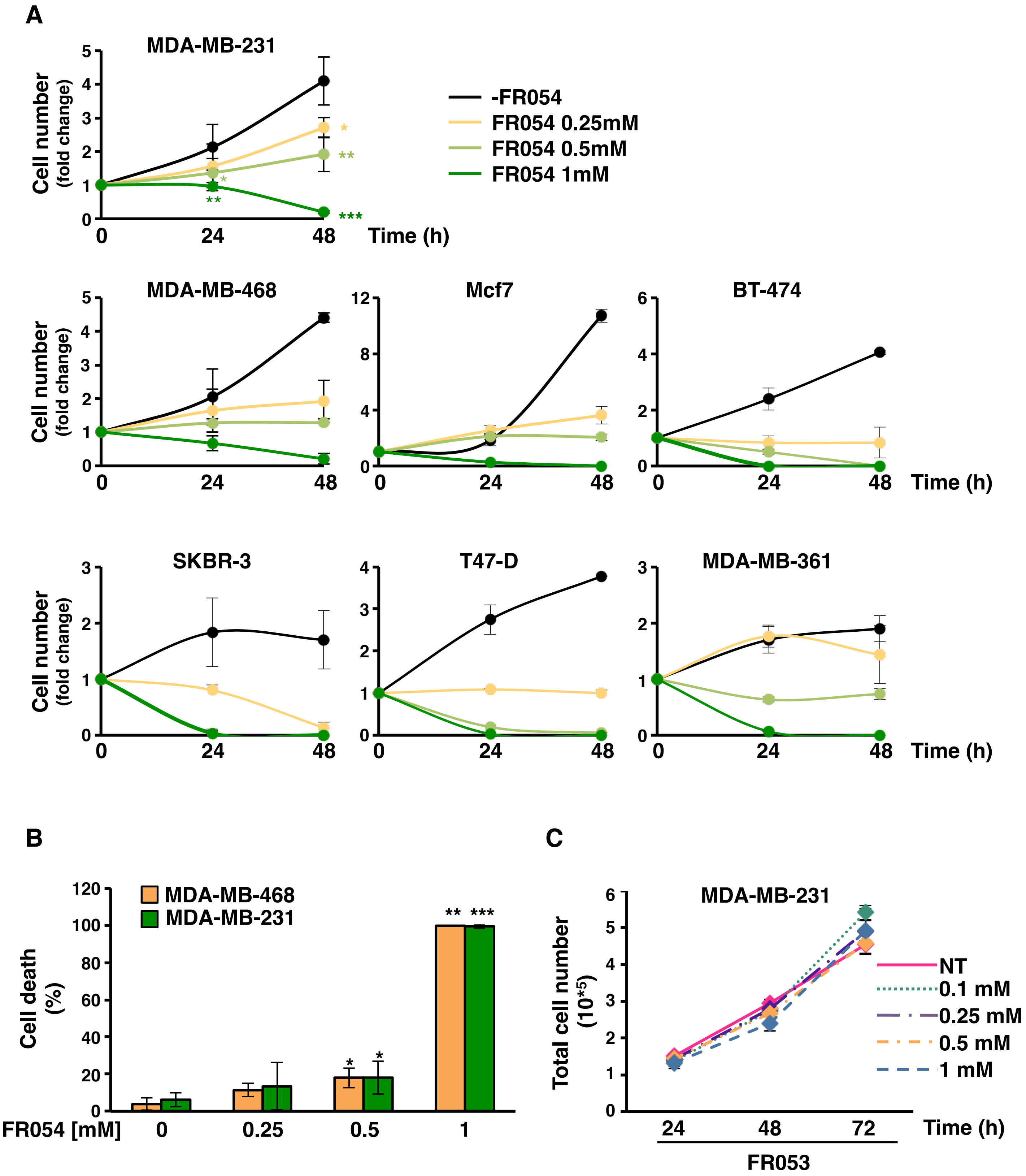

Supplementary Figure 3. FR054 treatment affects proliferation and viability of breast cells.

(A) Proliferation of breast cells upon treatment with different doses of FR054. (B) Cell death of MDA-MB-468 and MDA-MB-231 cells upon 72h of treatment with different doses of FR054. (C) Cell count of MDA-MB-231 cells upon treatment with different doses of FR053. All data represent the average ± s.d.; *p<0.05, **p<0.01, ***p<0.001, (Student's t-test; comparison with minus FR054); N=3.

A

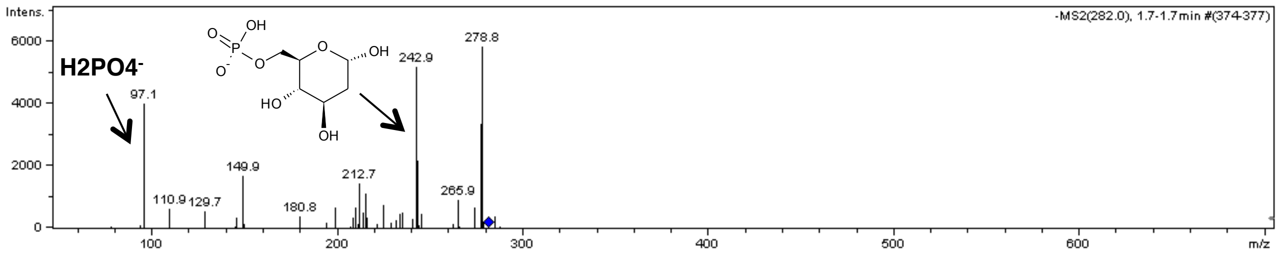

B

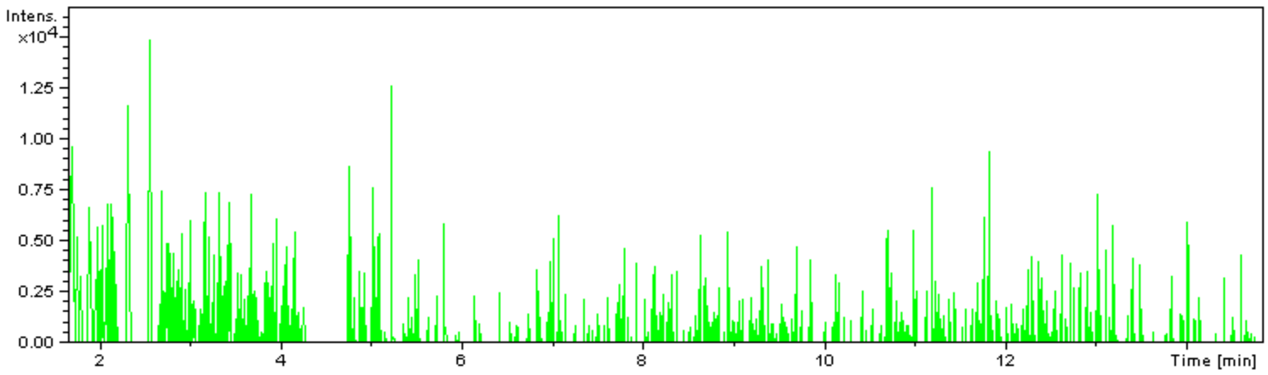

C

	Samples	FR054 (μM)	SD	Average	Ave.SD	FR053 (μM)	SD	Average	Ave.SD	FR051 (μM)	SD	Average	Ave.SD
1	Untreated	N.D.				N.D.				N.D.			
2	Untreated	N.D.				N.D.				N.D.			
3	Untreated	N.D.				N.D.				N.D.			
4	Treated	0,41	0,12	0,56	0,17	0,18	0,05	0,21	0,06	0,15	0,05	0,16	0,05
5	Treated	0,95	0,29			0,19	0,06			0,07	0,02		
6	Treated	0,32	0,1			0,25	0,08			0,25	0,07		

Supplementary Figure 4. Analysis of FR054 cell incorporation in MDA-MB-231 upon 24h treatment.

(A) The mass spectrum of intracellular FR051 in MDA-MB-231 cells by tandem mass spectrometry (MS/MS). (B) Extraction ion chromatogram for FR051 semiquantitative analysis is shown. The absence of in source fragmentation was verified by analyzing the samples in full scan mode and selectively monitoring both the phosphate neutral loss and the phosphate ion at m/z 79 in negative acquisition mode. No chromatographic peaks associated to in source phosphate loss were detected. (C) Quantitative analysis of FR054 and FR053, and semiquantitative analysis of FR051. Total amount of the three species is calculated by adding the average of the three forms identified into the cells: 0.925μM +/- 0.106μM s.d. N=3, each sample analyzed 3 times.

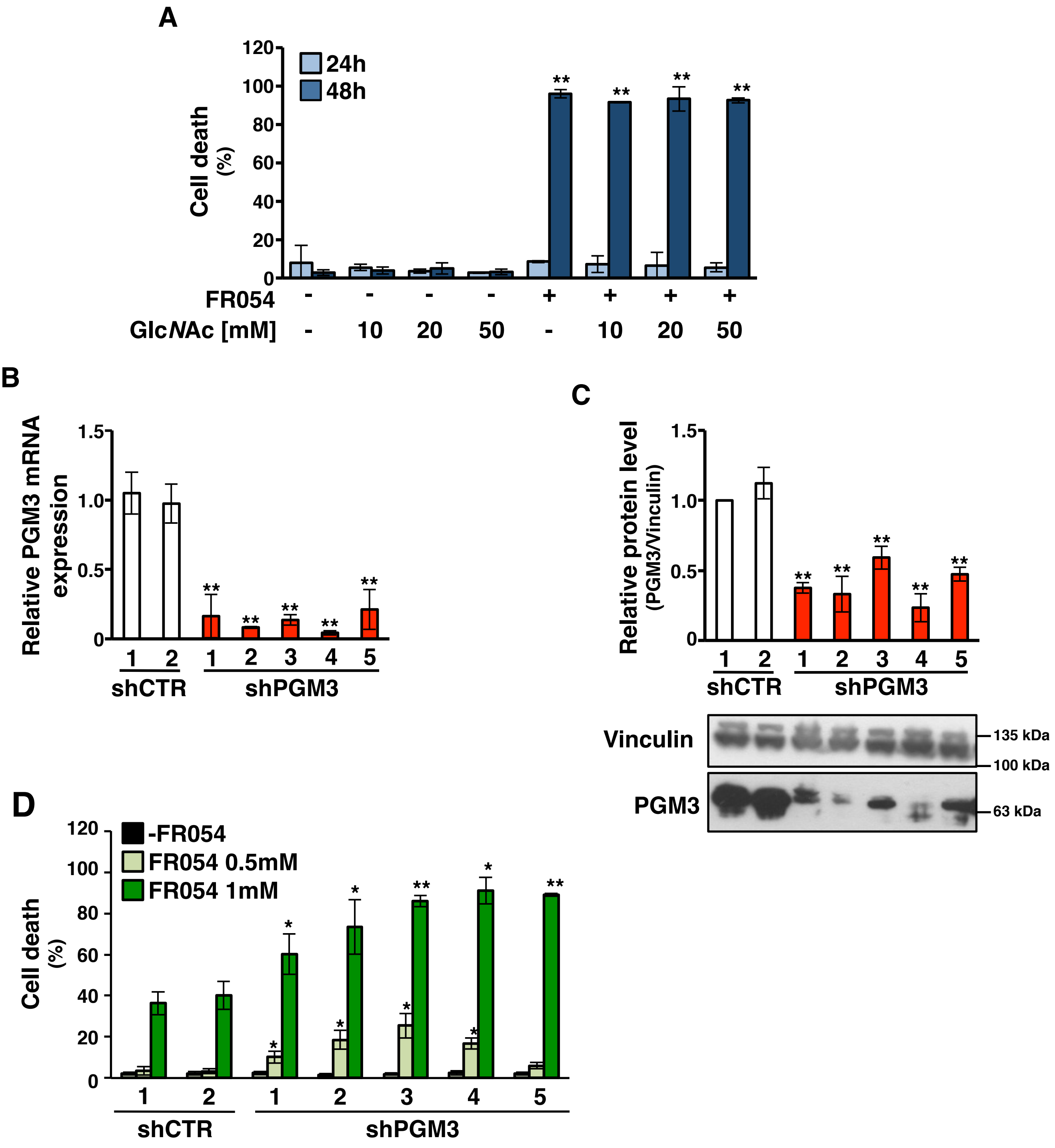

Supplementary Figure 5, related to Figure 3. FR054-induced apoptosis is not reverted by Glc*N*Ac treatment and increased in shPGM3 clones. (A) MDA-MB-231 cells were treated with 1mM FR054 for 24 and 48h or co-treated with increasing doses of Glc*N*Ac. Cell viability was evaluated after 24 and 48h of treatment by trypan blue exclusion assay. Data represent the average ± s.d.; **$p<0.01$, (Student's t-test; comparison with minus FR054); N=3. (B-C) mRNA (B) and protein (C) levels quantization in MDA-MB-231 stable clones shCTR and shPGM3. (D) Cell death determination by viable count of MDA-MB-231 stable clones shCTR and shPGM3 upon 48h treatment with FR054 0.5mM and 1mM. All data represent the average ± s.d.; *$p<0.05$, **$p<0.01$, (Student's t-test; comparison shPGM3 *vs.* shCTR); N=3.

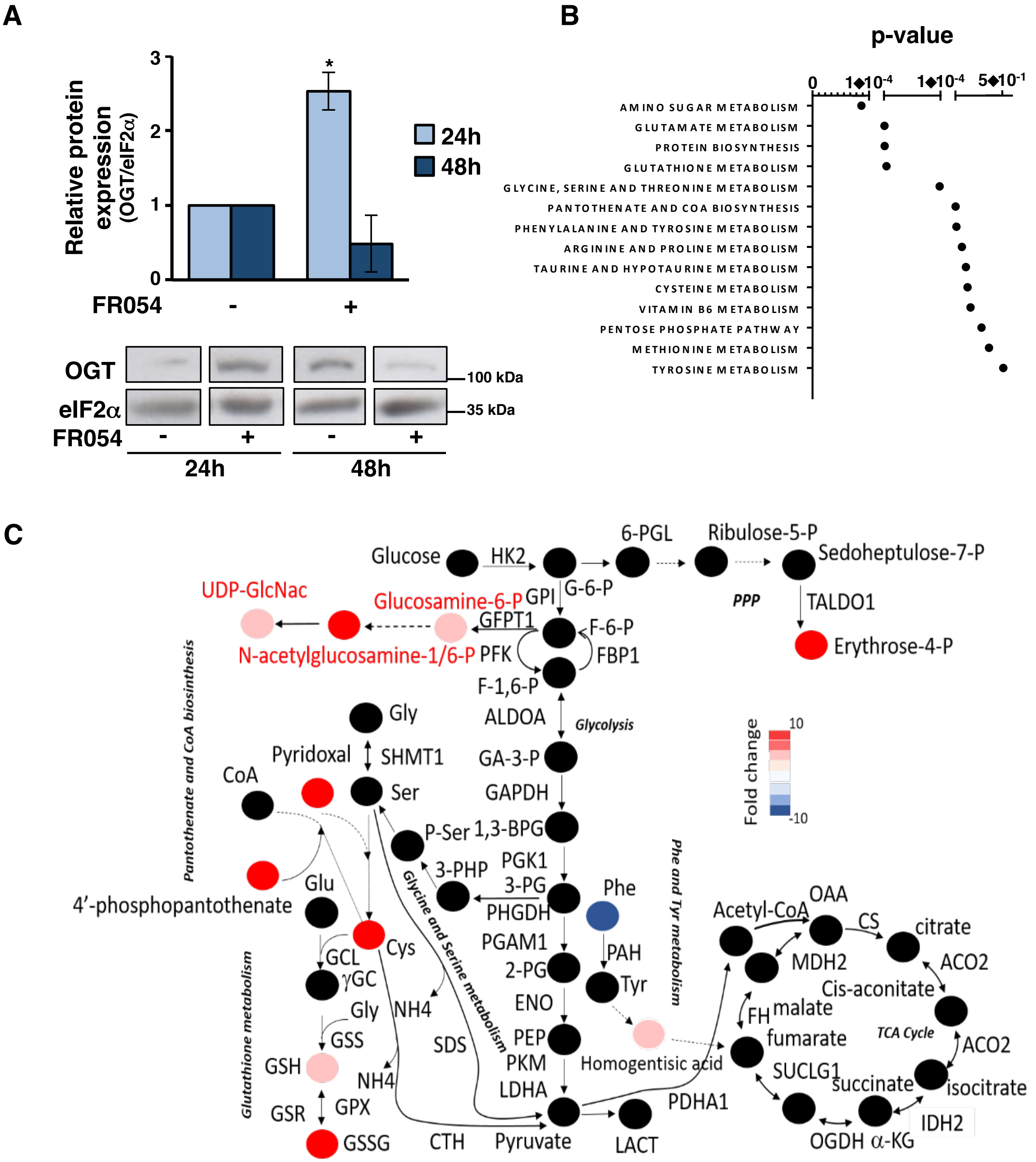

Supplementary Figure 6, related to Figure 4. FR054 treatment influences HBP flux and OGT protein.
(A) Total cell extracts from MDA-MB-231 cells treated for 24 and 48h with 1mM FR054 were subjected to Western Blot analysis in order to detect OGT protein by using specific antibody. A representative image of at least three independent experiments and relative band intensity quantitation is shown. Total eIF2α was used as loading control. Data represent the average ± s.d.; *$p<0.05$, (Student's t-test; comparison with minus FR054). N=3. (B) Graphic summary of metabolite set enrichment analysis for MDA-MB-231 cells treated with 1mM FR054. (C) Graphic visualization of the significantly deregulated metabolic pathways in MDA-MB-231 cells upon FR054 treatment as compared to untreated ones. The relative metabolites levels (average of replicates) are shown by a color-code, with the scale indicated; N=3.

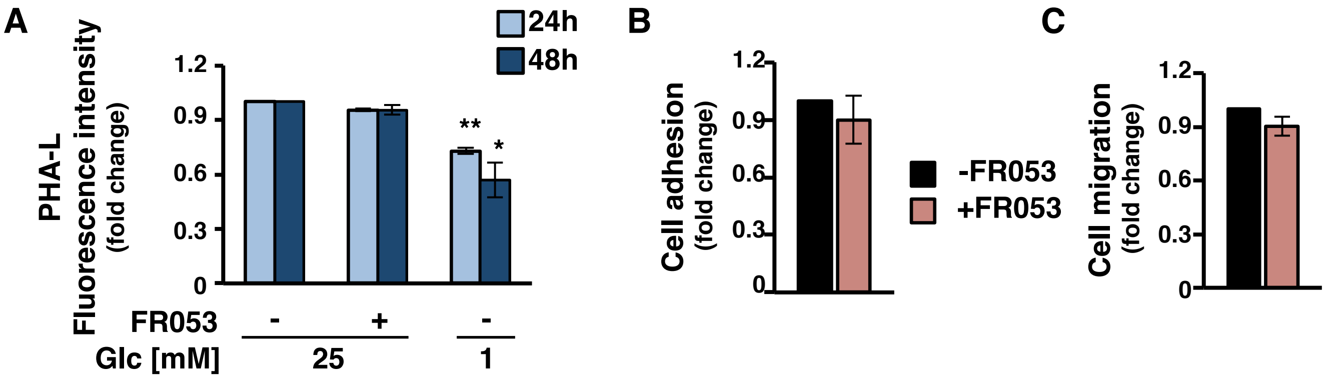

Supplementary Figure 7. FR053 does not affect MDA-MB-231 cell *N*-glycosylation, cell adhesion and migration.

(A) FACS analysis of membrane N-glycans in live cells, treated with FR053 or grown in 1mM glucose, stained with fluorochrome-conjugated PHA-L. MDA-MB-231-cell adhesion (B) and migration (C) upon 24h treatment with 250µM FR053. All data represent the average ± s.d.; *p<0.05, **p<0.01, (Student's t-test; comparison with minus FR053); N=3.

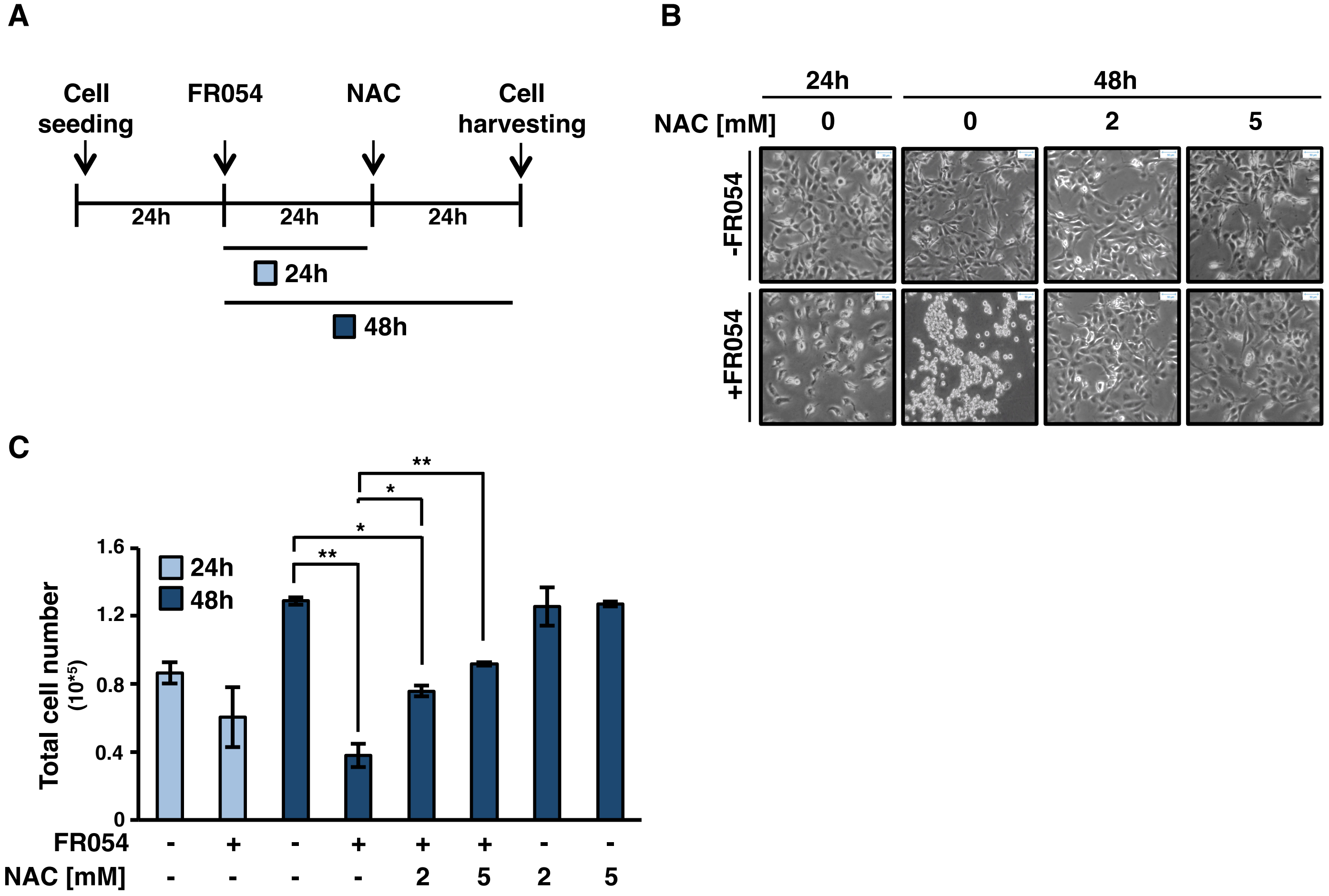

Supplementary Figure 8, related to Figure 5. NAC rescues the effects on cell morphology and proliferation induced by FR054 treatment in MDA-MB-231 cells.
(A) Schematic representation of the experimental workflow followed for the experiments with NAC. MDA-MB-231 were treated for 24h with FR054 and then incubated with two different doses of NAC (2 and 5mM).
According to the workflow described in (A), (B) phase contrast microscopy images were collected (4X magnification, 50µm scale) and (C) cell proliferation was evaluated upon 24 and 48h treatment with FR054.
All data represent the average ± s.d.; *p<0.05, **p<0.01 (Student's t-test); N=3.

Supplementary Table 1: Metabolites discriminating after 24h (Mann-Whitney-Wilcoxon Test $p<0.05$) FR054 treated (FR054) and untreated (CTR) MDA-MB-231 cells

		**Fold change**	**Peak intensity**								
**Metabolite**	**HMBD ID**	**FR054 vs CTR**	**MW_mean**	**CTR 1**	**CTR 2**	**CTR 3**	**FR054 1**	**FR054 2**	**FR054 3**	**Adduct**	**MW_mean**
Cysteine	HMDB00574	7.32	120.0126	886.6	1536.7	3932.9	8709.212	20259.58	17556.96	M-H	120.0126
Pyridoxal	HMDB01545	only FR054	166.0508	0	0	0	1431.35	4108.083	5388.777	M-H	166.0508
Homogentisic acid	HMDB00130	3.23	167.0353	6746.8	6523.1	5584.5	10736.01	22845.81	27384.2	M-H	167.0353
D-Erythrose 4-phosphate	HMDB01321	7.04	199.0015	1559.8	2640.4	0	6090.504	5234.237	18256.68	M-H	199.0015
D-4'-Phosphopantothenate	HMDB01016	14.06	298.0697	4196.4	3153.9	0	21738.84	38349.72	43280.53	M-H	298.0697
N-Acetyl-D-Glucosamine 6-Phosphate/(1P)	HMDB01062	16.67	300.0489	11848.8	6303	0	22119.01	53082.7	227341.6	M-H	300.0489
Glutathione	HMDB00125	2.26	306.0765	752984	802058.5	913806.4	1001998	1769556	2809531	M-H	306.0765
Uridine diphosphate-N-acetylglucosamine	HMDB00290	2.80	606.0749	81019.4	51058.7	0	86024.58	108883.9	174929.7	M-H	606.0749
Proline	HMDB00162	3.70	116.0706	7194.3	792.4	0	8343.753	12221.38	8968.034	M+H	116.0706
Creatine	HMDB00064	-4.17	154.0588	3829.6	5962.2	3180.2	1188.77	1924.513	0	M+Na	154.0588
Threonine	HMDB00167	-4.13	158.0214	36727.2	36024.8	27935	0	9651.282	14720.39	M+K	158.0214
Phenylalanine	HMDB00159	only CTR	188.0682	619.9	1547.8	3280.3	0	0	0	M+H	188.0682
Glucosamine 6-phosphate	HMDB01254	13.06	260.0534	2628	1662.3	0	10856.18	7505.729	37682.95	M+H	260.0534
N-Acetyl-glucosamine 1-phosphate/(6P)	HMDB01367	only FR054	302.0640	0	0	0	404513.7	389165.2	379403.9	M+H	302.0640
Oxidized glutathione	HMDB03337	12.76	635.1417	9188.5	0	0	48526.89	14672.6	54080.8	M+Na	635.1417
